# Streamlined modular synthesis of saframycin substructure via copper-catalyzed three-component assembly and gold-promoted 6-*endo* cyclization

**DOI:** 10.3762/bjoc.21.14

**Published:** 2025-01-28

**Authors:** Asahi Kanno, Ryo Tanifuji, Satoshi Yoshida, Sota Sato, Saori Maki-Yonekura, Kiyofumi Takaba, Jungmin Kang, Kensuke Tono, Koji Yonekura, Hiroki Oguri

**Affiliations:** 1 Department of Chemistry, Graduate School of Science, The University of Tokyo, 7-3-1 Hongo, Bunkyo-ku, Tokyo 113-0033, Japan; 2 Department of Applied Chemistry, School of Engineering, The University of Tokyo, FS CREATION, Mitsui LINK Lab Kashiwanoha 1, 6-6-2 Kashiwanoha, Kashiwa, Chiba, 227-0882, Japan; 3 Institute for Molecular Science (IMS), 5-1 Higashiyama, Myodaiji, Okazaki, Aichi, 444-8787, Japan; 4 RIKEN SPring-8 Center, 1-1-1 Kouto, Sayo, Hyogo 679-5148, Japan; 5 Japan Synchrotron Radiation Research Institute, 1-1-1 Kouto, Sayo, Hyogo, 679-5198, Japan; 6 Institute of Multidisciplinary Research for Advanced Materials, Tohoku University, 2-1-1 Katahira, Aoba-ku, Sendai 980-8577, Japan

**Keywords:** cascade reactions, copper-catalyzed three-component coupling, gold-mediated 6-*endo* hydroamination, tandem cyclizations, tetrahydroisoquinoline alkaloids

## Abstract

The integration of copper(I)-catalyzed three-component coupling with gold(I)-mediated 6-*endo* cyclization streamlines the rapid and modular assembly of the substructure of bis-tetrahydroisoquinoline (THIQ) alkaloids. The design of the key synthetic intermediate bearing a 2,3-diaminobenzofuran moiety allows both gold(I)-mediated regiocontrolled 6-*endo* hydroamination and temporary protection of nitrile and phenolic hydroxy groups. The synthetic strategy enabled the efficient synthesis of the substructure of saframycins bearing isoquinoline and THIQ units in just four steps from the modular assembly of the three components. We also found the unexpected involvement of a fluorescent intermediate in the cascade synthetic process.

## Introduction

**Figure 1:**
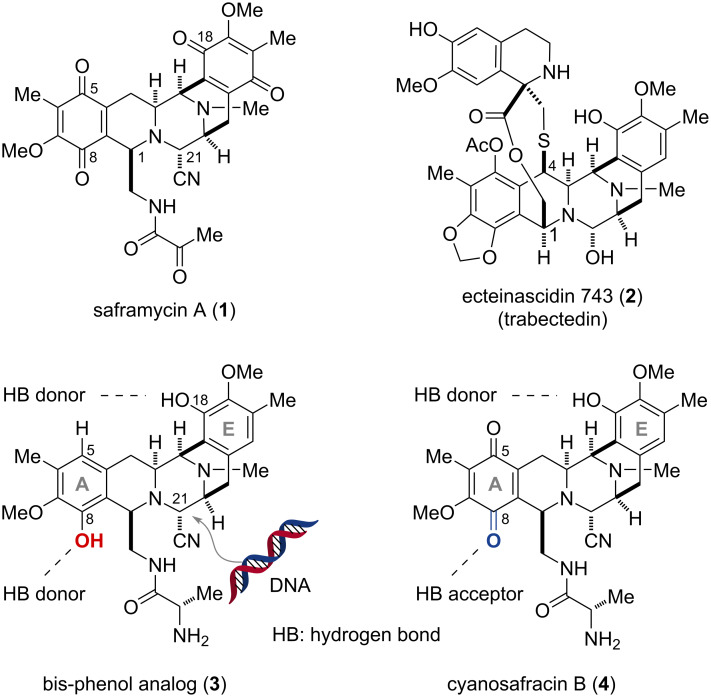
Representative bis-tetrahydroisoquinoline (THIQ) alkaloids and their analogues. Oxygen atoms on both the A- and E- rings serve as hydrogen bond (HB) donors/acceptors to facilitate DNA alkylation at C21.

The bis-tetrahydroisoquinoline (THIQ) alkaloid family represented by saframycin A (**1**) and ecteinascidin 743 (**2**) shares a complex penta- or hexacyclic core skeleton composed of two THIQ units (Figure 1) [1–5]. As proven by the clinical use of compound **2** for the treatment of malignant soft tissue sarcomas, the bis-THIQ alkaloid family exhibits potent antitumor activity, triggered by DNA alkylation [6–8]. The aminonitrile/hemiaminal at C21 generates an iminium cation while releasing a cyanide or a hydroxy group under physiological conditions. This iminium cation facilitates nucleophilic attack by guanine residues in the minor groove of the GC-rich region of the DNA double helix, leading to the formation of a reversible covalent bond [9–12]. In this process, the oxygen functional groups at the C8 and C18 positions of the core scaffold interact with the DNA bases through multipoint hydrogen bonds (HBs), allowing recognition of approximately three base pairs, predominantly 5’-GGC-3’ and 5’-GGG-3’ [12–13]. Notably, a bis-phenol type unnatural analog **3**, composed of the C5 deoxy A-ring bearing a phenolic hydroxy group at C8, presumably as a HB donor upon interaction with nucleic acids, exhibits superior DNA alkylation capability compared to the natural product, cyanosafracin B (**4**), bearing a para-quinone moiety at the left end [14]. Considering the relationships between the aromatic ring structures and DNA alkylating ability, we envisioned a modular and flexibly modifiable synthetic approach that would allow for the initial installation of lower oxidation state aromatic rings at the A- and E-rings of saframycins. Rational and systematic modification of both ends of the THIQ scaffolds would facilitate the development of reversible covalent DNA binders with tailored sequence preferences.

**Scheme 1:**
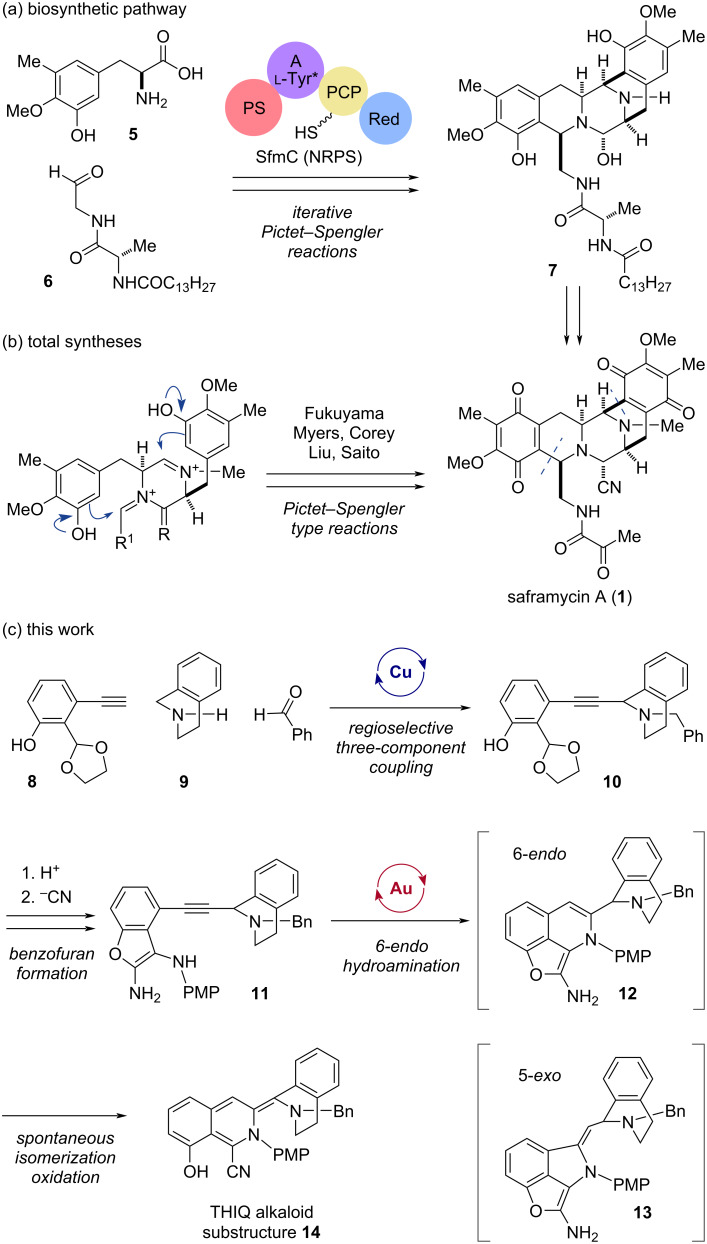
Strategies for the construction of the pentacyclic core scaffold of saframycin A (**1**). (a) Biosynthetic machinery catalyzed by an NRPS module SfmC. (b) Total syntheses utilizing the Pictet–Spengler-type reactions. (c) This work: streamlined modular assembly featuring copper(I)-catalyzed regiocontrolled three-component coupling (**8 → 10**), one-pot formation of the 2,3-diaminobenzofuran ring in the key intermediate **11**, and subsequent gold(I)-mediated regiocontrolled 6-*endo* hydroamination followed by cascade oxidative conversion and ring opening giving rise to the skeleton **14**.

Biosynthetically, the pentacyclic core scaffold of saframycin A (**1**) is assembled from two molecules of ʟ-tyrosine derivative **5** and peptidyl aldehyde **6** by non-ribosomal peptide synthetases (NRPS, Scheme 1a) [15–21]. The pivotal NRPS module, SfmC, catalyzes iterative regio- and stereoselective Pictet–Spengler (PS)-type cyclization to efficiently construct the pentacyclic intermediate **7**, as demonstrated in our previous study [15]. Following the pioneering total synthesis of saframycin A (**1**) by Fukuyama and co-workers taking advantage of the compatibility of phenolic hydroxy groups with PS-type cyclization [22], other groups led by Corey [23], Myers [24–25], Liu [26–27], and Saito [28] also efficiently exploited PS-type reactions to accomplish the total synthesis of saframycin A (**1**) (Scheme 1b) [3–5,29–41]. However, PS-type reactions impose constraints due to the necessity of electron-donating groups on the aromatic rings to facilitate S_E_Ar reactions. These constraints have stimulated interest in exploring an alternative synthetic approach to achieve greater structural diversification [42].

In this study, to overcome the synthetic limitations inevitably imposed by reliance on the biomimetic PS reactions, we sought to develop a de novo synthetic process that is independent of the substituents on the aromatic rings at both ends (Scheme 1c). To achieve more rapid synthesis and flexible structural diversification of the alkaloidal scaffolds, we conceived a streamlined modular synthetic strategy involving the cascading assembly of the left THIQ segment. A concise modular synthetic process was developed to construct the substructure **14** of saframycin A (**1**), featuring copper(I)-catalyzed three-component coupling, and subsequent tandem 2,3-diaminobenzofuran formation, followed by gold(I)-promoted 6-*endo* cyclization between the internal alkyne and 2,3-diaminobenzofuran moieties with spontaneous transformations into the left THIQ segment in **14**.

## Results and Discussion

As outlined in our modular synthetic approach (Scheme 1c), the copper(I)-catalyzed three-component coupling of alkyne **8**, THIQ segment **9**, and benzaldehyde would enable convergent assembly of the building blocks to produce **10** [43–46]. Removal of the cyclic acetal in **10** followed by Strecker-type conversion leading to an α-amino nitrile would enable tandem intramolecular cyclization with phenol to form 2,3-diaminobenzofuran **11**. The subsequent gold(I)-mediated intramolecular 6-*endo* hydroamination of **11** would construct the left THIQ ring to furnish the substructure **14** of saframycin A (**1**) [47–51]. To selectively promote the desired 6-*endo* cyclization (**11 → 12**) over the competing 5-*exo* pathway leading to **13**, we strategically designed 2,3-diaminobenzofuran **11** as a suitably functionalized cyclization precursor for the gold(I)-promoted hydroamination, considering the following three factors. Firstly, the appropriate trajectory of the secondary amino group in the benzofuran moiety is expected to facilitate the 6-*endo*-*dig* cyclization to the distant sp-carbon on the alkyne, as demonstrated by Fujii and Ohno in their total synthesis of (−)-quinocarcin [52–53]. Secondly, the 6-*endo*-cyclized product **12**, bearing the furan-conjugated isoquinoline-type framework, is predicted to be thermodynamically more stable than its 5-*exo* counterpart **13**. Thirdly, the 2,3-diaminobenzofuran would be utilized as a temporary protecting group for both the phenolic hydroxy group and the nitrile moiety. These functional groups are necessary for the aromatic A-ring to interact with DNA and for synthetic manipulation to install the C1 sidechain for saframycins, respectively [14,39].

**Scheme 2:**
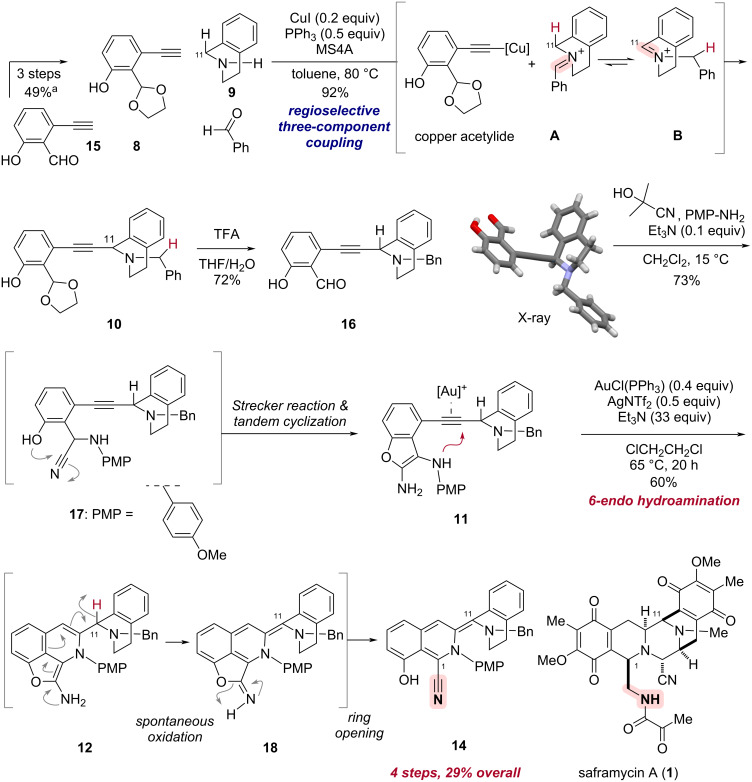
Streamlined synthesis of the substructure **14** for saframycins **1** within just four steps in overall 29% yield. This concise modular synthetic process involves copper(I)-catalyzed three-component coupling, sequential Strecker reaction and cyclization to form the 2,3-diaminobenzofuran moiety, and gold(I)-promoted cascade sequence via 6-*endo* hydroamination, spontaneous dehydrogenative oxidation followed by ring opening. Carbon numbering was assigned in accordance with natural products. The structure of **16** was determined by small crystal analysis using an X-ray free-electron laser (XFEL) [54–55]. ^a^See Supporting Information File 1 for details.

The alkyne segment **8** was prepared by protecting group manipulations in three steps from the known starting material, 2-ethynyl-6-hydroxybenzaldehyde (**15**), which can be readily synthesized from commercially available 1-bromo-3-fluorobenzene (see Scheme 2 and Supporting Information File 1 for details). Copper(I)-catalyzed three-component coupling reaction of alkyne **8**, THIQ **9**, and benzaldehyde, proceeded with exquisite control of regioselectivity to afford **10** in an excellent yield of 92% [43–46]. This efficient cascade reaction involves an in situ generation of the iminium cation **A** followed by isomerization to the thermodynamically more stable iminium cation **B**. Subsequent nucleophilic attack of a copper acetylide enabled regioselective C–C bond formation at the C11 position. After removal of the cyclic acetal, the structure of **16** was confirmed by serial X-ray crystallography using an X-ray free-electron laser (XFEL) [deposition number CCDC 2352718) [54–55].

We then performed a Strecker-type reaction on the aldehyde **16** to construct an α-aminonitrile **17**. To our delight, the key intermediate, 2,3-diaminobenzofuran **11**, was obtained in one-pot, presumably via generation of the aminonitrile **17** and subsequent nucleophilic attack of the phenolic hydroxy group to form the five-membered ring. Our efforts to optimize this one-pot sequence led to the best results, affording **11** in 73% isolated yield, when acetone cyanohydrin and a catalytic amount of triethylamine were used in dichloromethane with careful control of the reaction temperature at 15 °C (Table S2, Supporting Information File 1).

With the 2,3-diaminobenzofuran **11** in hand as the designed cyclization precursor, we explored the construction of the left isoquinoline ring via gold(I)-mediated 6-*endo* hydroamination (Scheme 2). Treatment of **11** with a cationic gold complex, generated in situ from AuCl(PPh_3_) and AgNTf_2_ [47–49,56], with an excess amount of triethylamine in 1,2-dichloroethane at 65 °C, resulted in the intended regiocontrolled hydroamination. The resulting 6-*endo* cyclization product **12** could not be isolated under these conditions, and instead, the unexpected formation of fluorescent transient intermediate **18** was observed. Dehydrogenative oxidation of the 6-*endo*-cyclized product **12** with transpositions of the double bonds conjugated to the enamine moiety, would afford the corresponding imidate **18** with incorporation of an extended conjugation system. The oxidation-labile nature of the corresponding C11 position in **12** is consistent with the low bond dissociation energies (BDEs) at both the α-position of the nitrogen, as shown in Supporting Information File 1, Figure S2 [57], and the benzylic position on the THIQ ring [58]. Indeed, termination of the reaction just after 90 minutes instead of 20 h resulted in the isolation of the fluorescent intermediate **18** in 55% yield (Scheme S1, Supporting Information File 1). Even with the use of both degassed solvent and light-shielding flask, the oxidative conversion of **12** to the imidate **18** proceeded smoothly. Notably, the transient intermediate **18**, exhibiting a sky-blue fluorescence, further underwent a ring opening to afford the tetracyclic **14** with the simultaneous regeneration of both a nitrile and a phenol moiety. The final step is assumed to be facilitated by the release of the ring distortion of the benzofuran system. Overall, triggered by the gold(I)-promoted 6-*endo* hydroamination between the 2,3-diaminobenzofuran and the alkyne in **11**, dehydrogenative oxidation of the resulting **12** to form a fluorescent intermediate **18**, and subsequent ring opening allowed a streamlined one-pot access to the substructure of THIQ alkaloids **14** in a good yield of 60% from **11**. The structure of the resulting **14** was elucidated through comprehensive two-dimensional NMR spectroscopy, complemented by NOE measurements (Figures S20 to S25, Supporting Information File 1). A notable feature of this cascade process is the temporary protection of the C≡N triple bond, nitrile in the key intermediate **11**, by the 2,3-diaminobenzofuran group. This facilitates the site-selective activation of the alkyne triple bond by the gold complex and the silver salt, to efficiently achieve the 6-*endo* cyclization and subsequent conversions.

**Figure 2:**
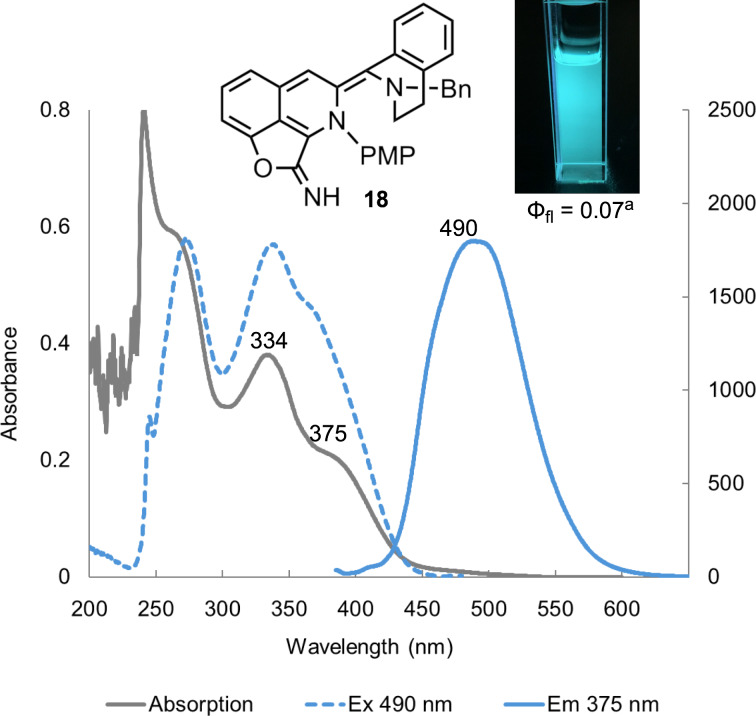
UV–vis absorption (gray solid line), the emission spectrum (blue solid line), and the corresponding excitation spectrum (blue dashed line) of the imidate **18** in CHCl_3_ (*c* = 100 μM). ^a^Quantum yield (Φ_fl_, excited at 375 nm): 0.07 (*c* = 10 μM). Emission image was captured under UV light (365 nm, *c* = 100 μM in CHCl_3_).

During the development of this cascade synthesis process, we serendipitously discovered the involvement of a sky-blue fluorescent transient intermediate **18** (Figure 2). We therefore investigated the optical properties of **18** in CHCl_3_ (*c =* 100 μM) by measuring its UV–vis absorption spectrum as well as its excitation and emission spectra. The UV–vis spectrum of **18** showed two absorption peaks at 334 nm and around 375 nm (gray solid line). When excited at 375 nm, the emission spectra of **18** displayed a relatively broad peak with a maximum around 490 nm (blue solid line). The excitation spectra of **18** (blue dashed line), corresponding to an emission at 490 nm, was also recorded (Figure S3, Supporting Information File 1 for details). Despite the modest emission quantum yield (Φ_fl_ = 0.07, excited at 375 nm), the chromophore of the pentacyclic intermediate **18** suggests a potential as a fluorescent probe.

## Conclusion

In summary, we have developed a novel synthetic approach for the efficient construction of the substructure of saframycin A (**1**). Our strategy streamlines the three key transformations: copper(I)-catalyzed regiocontrolled three-component assembly of alkyne **8**, THIQ segment **9**, and benzaldehyde to yield **10**, followed by tandem Strecker reaction and intramolecular cyclization to form 2,3-diaminobenzofuran **11**. Subsequent gold(I)-mediated 6-*endo* hydroamination of **11** leads to the formation of the left isoquinoline ring and ultimately the substructure of THIQ alkaloids **14**. This synthetic approach surpasses the limitations of Pictet–Spengler (PS)-type biomimetic reactions, offering greater flexibility for structural diversification at both the aromatic ends for future exploration. The THIQ alkaloids substructure **14** was efficiently synthesized in only four steps from the modular assembly of the three simple segments.

A notable feature of our approach is the temporary protection of the nitrile and phenolic hydroxy groups by the 2,3-diaminobenzofuran moiety, which facilitates efficient activation of the alkyne triple bond and allows precise control of the chemo- and regioselectivities for the assembly of the left isoquinoline substructure. The unexpected discovery of the fluorescent intermediate **18** adds an intriguing dimension to our current synthetic investigation and suggests potential avenues for the development of fluorescent probes based on the bis-THIQ alkaloidal scaffold. Further efforts to develop a concise and modular synthetic process for saframycins are currently underway.

## Supporting Information

File 1 The experimental procedures and characterization data, including copies of NMR spectra and X-ray crystallographic analyses.

## Data Availability

All data that supports the findings of this study is available in the published article and/or the supporting information of this article.
