# Supplementary material for: Streamlined modular synthesis of saframycin substructure via copper-catalyzed three-component assembly and gold-promoted 6-endo cyclization
Source: Beilstein J Org Chem. 2025 Jan 28;21:226–33. doi: 10.3762/bjoc.21.14 (PMC11789684; doi:10.3762/bjoc.21.14)
Supplement: File 1 — The experimental procedures and characterization data, including copies of NMR spectra and X-ray crystallographic analyses. [file Beilstein_J_Org_Chem-21-226-s001.pdf]

BEILSTEIN JOURNAL OF ORGANIC CHEMISTRY

Supporting Information

for

Streamlined modular synthesis of saframycin substructure via copper-catalyzed three-component assembly and gold-promoted 6-*endo* cyclization

Asahi Kanno, Ryo Tanifuji, Satoshi Yoshida, Sota Sato, Saori Maki-Yonekura, Kiyofumi Takaba, Jungmin Kang, Kensuke Tono, Koji Yonekura and Hiroki Oguri

Beilstein J. Org. Chem. **2025,** *21,* 226–233. doi:10.3762/bjoc.21.14

The experimental procedures and characterization data, including copies of NMR spectra and X-ray crystallographic analyses

License and Terms: This is a supporting information file under the terms of the Creative Commons Attribution License (https://creativecommons.org/licenses/by/4.0). Please note that the reuse, redistribution and reproduction in particular requires that the author(s) and source are credited and that individual graphics may be subject to special legal provisions.
The license is subject to the *Beilstein Journal of Organic Chemistry* terms and conditions: (https://www.beilstein-journals.org/bjoc/terms)

Table of contents

Supplementary tables and figures S2

General methods and materials S6

Synthetic procedures S7

^{1}H, ^{13}C NMR spectra S12

X-ray crystallographic analysis S23

References S26

Supplementary tables and figures

Table S1. Optimization of three-component coupling to synthesize **10**.[a]

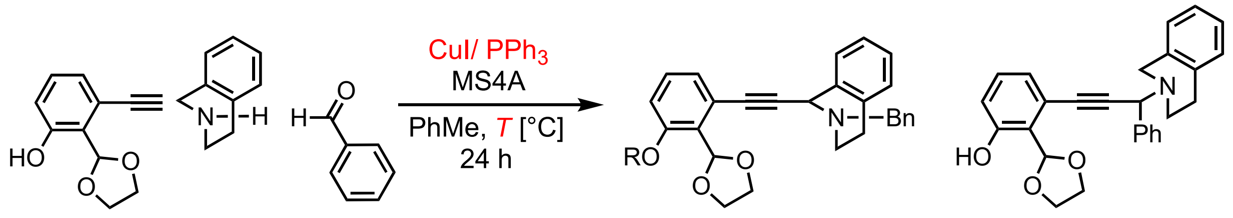

8 **9** **10** **S4**

entry	*T* [°C]	CuI/ PPh_3 [eq.]	yield of **10** [%] [b]	yield of **S4** [%] [b]
1	50	0.2 / 0	48	9
2	50	0.2/ 0.2	46	4
3	50	0.2/ 0.5	48	0
4	80	0.2/ 0.5	**53**	0
5	20	0.2/ 0.5	5	2

[a] General reaction conditions: Alkyne **8** (105 µmol, 1.0 eq.), THIQ **9** (1.0 eq.), benzaldehyde (1.0 eq.), MS4A (200 mg), toluene (0.05 M), 24 h.

[b] Determined by ^{1}H NMR spectroscopy of the unpurified crude reaction mixture based on qNMR using triphenylmethane (purchased from FUJIFILM Wako, for qNMR grade, 0.20 eq.) as an internal standard.

Table S2. Optimization of tandem reaction of **16** to synthesize **11**.[a]

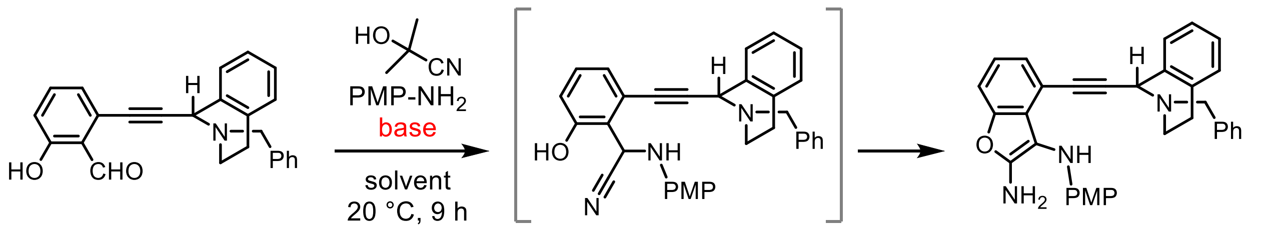

16 **17** **11**

entry	base	solvent	NMR yield of **11** (%) [b]
1	Et_3N	$CHCl_3$	66
2	(i-Pr)$_2$NEt	$CHCl_3$	47
3	N-methylmorpholine	$CHCl_3$	33
4	DABCO	$CHCl_3$	51
5	pyridine	$CHCl_3$	not detected
6	Et_3N	THF	27
7	Et_3N	DCM	23
8	Et_3N	1,2-DCE	21
9	Et_3N	MeCN	10
10	Et_3N	toluene	2

[a] General reaction conditions: Aldehyde **16** (109 µmol, 1.0 eq.), acetone cyanohydrin (10 eq.), *p*-Anisidine (1.3 eq.), base (5.0 eq.), solvent (0.07 M), 20 °C, 9 h.
[b] Determined by ^{1}H NMR spectroscopy of the unpurified crude reaction mixture based on qNMR using triphenylmethane (purchased from FUJIFILM Wako, for qNMR grade, 0.10 eq.) as an internal standard.

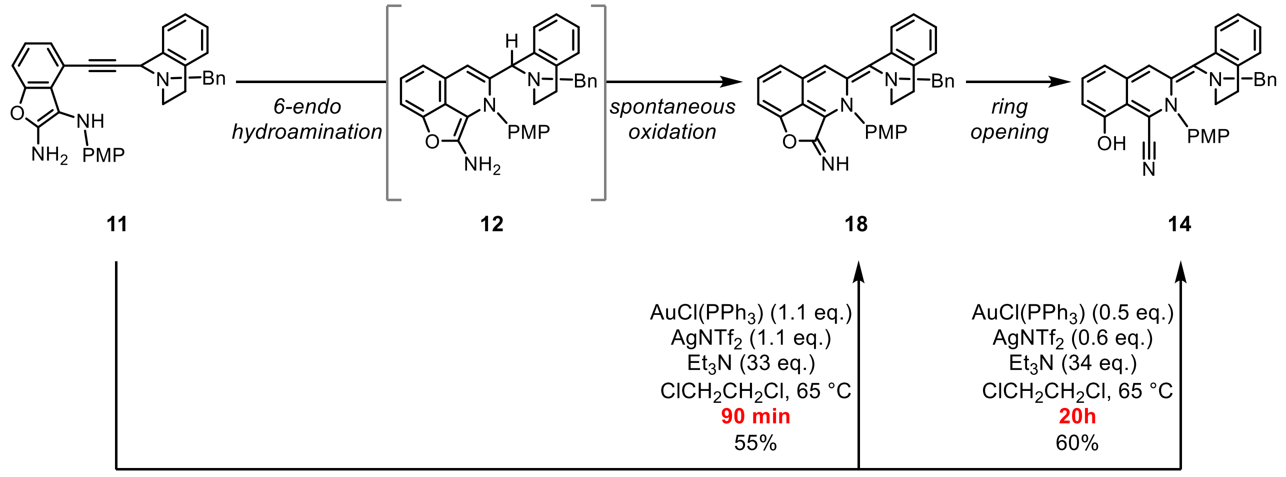

Figure S1. XFEL (I)-promoted 6-*endo* cyclization and following reactions to yield **12**, **18**, and **14**.

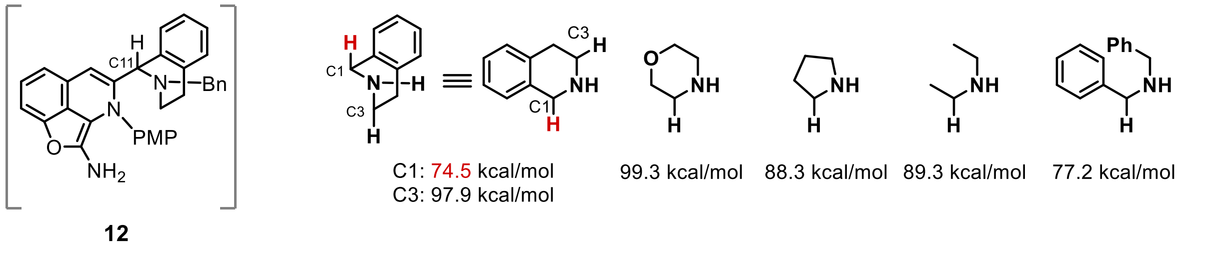

Figure S2. Calculated bond dissociation energies (BDEs) of the C–H bonds similar to the C11 position of compound **12** as depicted in the reference figure.[1]

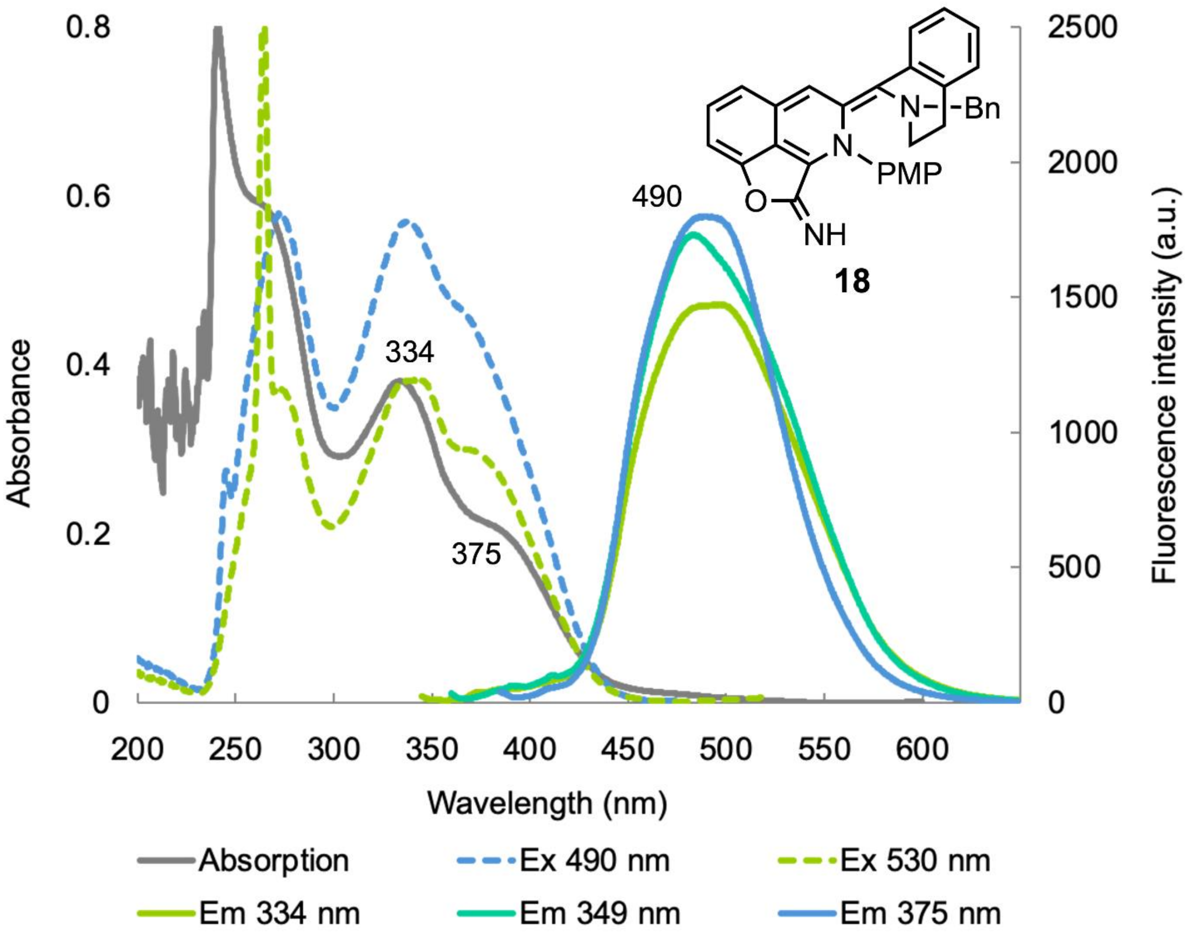

Figure S3. UV–vis absorption spectrum (gray solid line), emission spectra (solid lines), and the corresponding excitation spectra (dashed lines) of imidate **18** in $CHCl_3$ (c = 100 μM).

General methods and materials

All the reactions were performed under nitrogen atmosphere unless otherwise specified. All the reactions requiring heating were conducted in a heating mantle, and reaction temperatures are reported as the temperature of the heat transfer medium surrounding the vessel. Reactions were monitored by thin layer chromatography using Merck Millipore TLC silica gel F254 plates (0.21–0.27 mm), which were visualized using UV light, phosphomolybdic acid (PMA) stain, PMS stain, *p*-anisaldehyde stain, or ninhydrin stain. Flash column chromatography was performed using Wakosil® 60 (38–63 µm). The medium-pressure liquid chromatography (MPLC) purifications were performed on a Biotage® Isolera or Biotage® Selekt. NMR spectra were recorded on JEOL ECS 400 (^{1}H/400 MHz, ^{13}C{^{1}H}/100 MHz) spectrometer. Chemical shifts were quoted in parts per million (ppm) from chloroform as an internal standard of 7.26 and 77.16 for ^{1}H and ^{13}C NMR, respectively. Data for ^{1}H NMR were reported as follows: chemical shift (number of hydrogens, multiplicity, coupling constant). Multiplicity is abbreviated as follows: s (singlet), d (doublet), t (triplet), q (quartet), m (multiplet), br (broad). ESI-Mass spectra were recorded on a Bruker micrOTOF-II. At the SACLA XFEL facility, X-ray diffraction data of small crystals of intermediate **16** were collected on beamline BL2 as described.[2] The fluorescent spectra were recorded on JASCO FP-8500. The absolute quantum yield of intermediate **18** was quantified using a Hamamatsu Photonics C9920-02G absolute photoluminescence quantum yield spectrometer.

***Cyanide source is seriously hazardous and need to be handled in a fume hood with extra care.**

Commercial solvents and reagents were used as received with the following exceptions. Chloroform was purified by filtering through aluminum oxide (1st grade Wako, contains 0.3–1.0% ethanol). Copper(I) iodide was purified by recrystallization from aqueous solution of potassium fluoride. $MgSO_4$ and MS 4Å were activated using a heat-gun in vacuo right before its use.

Synthetic procedures

Compound S2

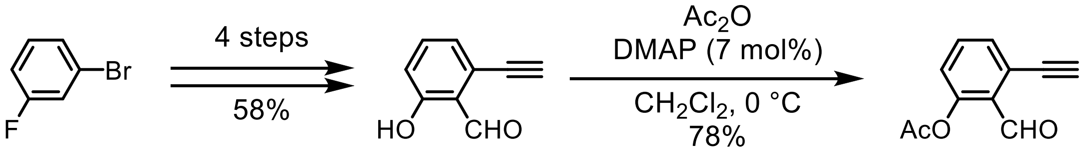

To a solution of **15**[3] (22.3 mg, 153 µmol, 1.0 equiv) in CH_2Cl_2 3 mL (0.05 M) were added triethylamine (51.1 µL, 367 µmol, 2.4 equiv), acetic anhydride (17.3 µL, 183 µmol, 1.2 equiv), and DMAP (1.29 mg, 10.6 µmol, 0.07 equiv). The reaction mixture was stirred at 0 °C for 20 min. Additional acetic anhydride (10.0 µL, 106 µmol, 0.69 equiv) was added and the mixture was kept stirring for 10 min. The resulting reaction mixture was concentrated in vacuo, and the residue was purified by silica-gel column chromatography (EtOAc/hexane) to afford **S2** (22.6 mg, 120 µmol, 78%).

^{1}H NMR (400 MHz, $CDCl_3$) δ 10.51 (1H, d, J = 0.9 Hz), 7.56–7.53 (2H, m), 7.12 (1H, dd, J = 7.6, 2.5 Hz), 3.50 (1H, s), 2.38 (3H, s); ^{13}C NMR (100 MHz, $CDCl_3$) δ 189.8, 169.4, 150.0, 134.4, 132.1, 128.6, 127.7, 124.8, 85.6, 78.9, 21.0; HRMS (ESI, *m*/*z*): $[M+H]^+$ calcd. for $C_{11}H_8NaO_3$, 211.0366; found 211.0406.

Compound S3

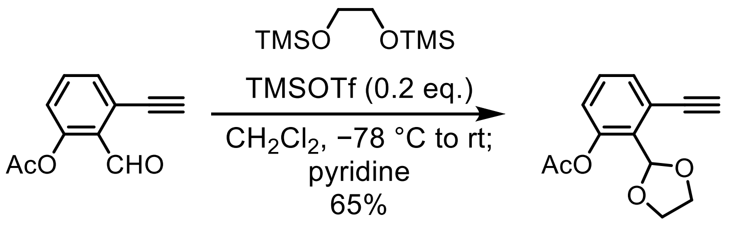

To a solution of 1,2-bis(trimethylsiloxy)ethane (2.26 mL, 9.22 mmol, 2.4 equiv) and **S2** (723 mg, 3.84 mmol, 1.0 equiv) in CH_2Cl_2 (12.8 mL, 0.3 M) was added TMSOTf (139 µL, 768 µmol, 0.2 equiv) at −78 °C. After being stirred at −78 °C for 3 h, the resulting mixture was stirred at ambient temperature for 5 min and quenched by treatment with pyridine (311 µL, 3.84 mmol, 1.0 equiv). The resulting reaction mixture was then poured into a cold saturated aqueous solution of $NaHCO_3$. The aqueous phase was extracted three times with CH_2Cl_2, and the combined organic layers were dried over Na_2SO_4, filtered, and concentrated in vacuo. The crude residue was recrystallized from toluene to afford **S3** (584 mg, 2.51 mmol, 65%) as a beige crystalline solid.

^{1}H NMR (400 MHz, $CDCl_3$) δ 7.44 (1H, dd, J = 7.8, 1.4 Hz), 7.33 (1H, t, J = 7.8 Hz), 7.06 (1H, dd, J = 8.0, 1.1 Hz), 6.24 (1H, s), 4.20–4.17 (2H, m), 4.05–4.01 (2H, m), 3.34 (1H, s), 2.28 (3H, s); ^{13}C NMR (100 MHz, $CDCl_3$) δ 169.1, 149.8, 131.9, 130.8, 130.0, 124.8, 123.8, 100.7, 82.6, 80.6, 66.0, 20.9; HRMS (ESI, *m*/*z*): $[M+H]^+$ calcd. for $C_{13}H_{12}NaO_4$, 255.0628; found 255.0673.

Compound **8**

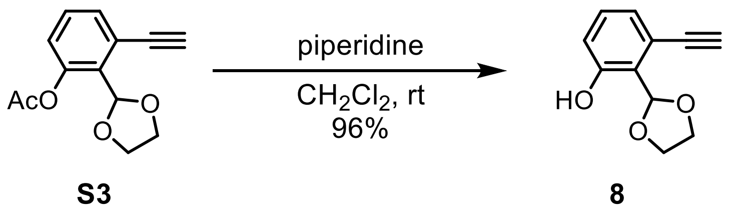

To a stirred solution of **S3** (282 mg, 1.22 mmol, 1.0 equiv) in CH_2Cl_2 12 mL (0.1 M) was added piperidine (360 µL, 3.65 mmol, 3.0 equiv) at room temperature. The mixture was stirred for 1 d before concentration in vacuo. The residue was dissolved in CH_2Cl_2 and washed with saturated aqueous solution of NH_4Cl and brine, dried over Na_2SO_4, and concentrated in vacuo. The crude residue was purified by silica-gel column chromatography (EtOAc/hexane) to afford **8** (222 mg, 1.17 mmol, 96%) as a white crystalline solid.

^{1}H NMR (400 MHz, $CDCl_3$) δ 8.48 (1H, s), 7.21 (1H, t, J = 7.8 Hz), 7.10–7.06 (1H, m), 6.92–6.89 (1H, m), 6.26 (1H, s), 4.29–4.20 (2H, m), 4.14–4.06 (2H, m), 3.27 (1H, s); ^{13}C NMR (100 MHz, $CDCl_3$) δ 156.9, 130.8, 125.3, 122.9, 120.3, 118.9, 104.3, 81.9, 80.8, 65.1; LRMS (ESI, *m*/*z*): $[M+H]^+$ calcd. for $C_{11}H_{11}O_3$, 191.07; found 191.21.

Compound **10**

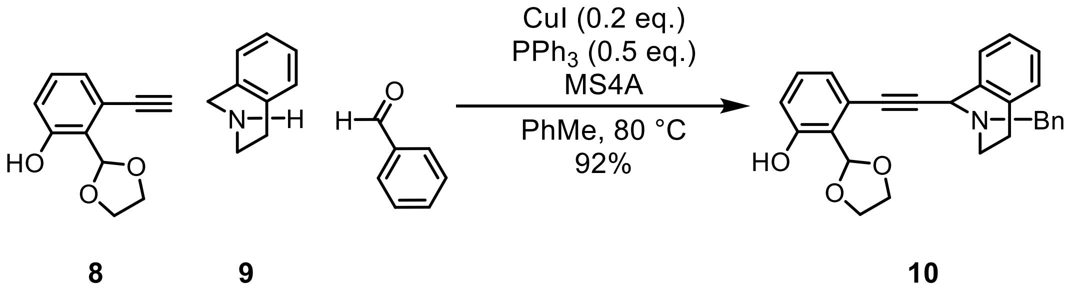

A mixture of **8** (218 mg, 1.15 mmol, 1.0 equiv), MS 4Å (901 mg), CuI (43.6 mg, 229 µmol, 0.20 equiv) and triphenylphosphine (152 mg, 581 µmol, 0.50 equiv) in toluene (11 mL, 0.1 M) was subjected to argon bubbling for 5 min, and then treated with benzaldehyde (117 µL, 1.15 mmol, 1.0 eq.) and 1,2,3,4-tetrahydroisoquinoline **9** (145 µL, 1.15 mmol, 1.0 equiv). After stirring at 80 °C for 24 h, the reaction mixture was cooled to room temperature and then quenched by adding SiliaMetS® TAAcONa (296 mg, 1.18 mmol, 1.0 equiv, SiliCycle) as a copper scavenger. After being stirred for 10 min, the solution was filtered through a pad of Celite®, and the solid residue was washed with chloroform. After concentration in vacuo, the crude residue was purified by silica-gel column chromatography (EtOAc/hexane) to afford **10** (433 mg, 1.05 mmol, 92%) as an amorphous brown solid.

^{1}H NMR (400 MHz, $CDCl_3$) δ 8.53 (1H, s), 7.51 (2H, d, J = 6.9 Hz), 7.37 (2H, dd), 7.33–7.28 (2H, m), 7.23–7.15 (4H, m), 7.05 (1H, d, J = 7.6 Hz), 6.88 (1H, d, J = 8.2 Hz), 6.13 (1H, s), 4.84 (1H, s), 4.21–4.15 (2H, m), 4.02–3.86 (4H, m), 3.09–3.06 (2H, m), 2.89–2.80 (2H, m); ^{13}C NMR (100 MHz, $CDCl_3$) δ 156.8, 138.4, 135.5, 134.1, 130.6, 129.3, 129.1, 128.4, 127.8, 127.3, 127.0, 125.9, 124.5, 124.0, 119.6, 118.0, 104.4, 92.6, 84.3, 64.9, 64.8, 59.7, 54.6, 46.0, 29.2; HRMS (ESI, *m*/*z*): $[M+H]^+$ calcd. for $C_{27}H_{26}NO_3$, 412.1908; found 412.1905.

Compound **16**

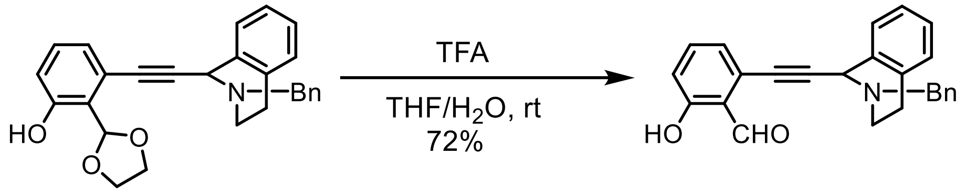

10 **16**

To a solution of **10** (433 mg, 1.05 mmol, 1.0 equiv) in THF (6.0 mL) was added water (6.0 mL) and trifluoroacetic acid (TFA) (6.0 mL, 78.4 mmol, 75 equiv) and stirred at 20 °C for 20 h. The resulting reaction mixture was basified with aqueous NaOH solution and saturated aqueous solution of $NaHCO_3$ to pH 8. The mixture was extracted three times with chloroform, and the combined organic layers were washed with brine, dried over Na_2SO_4, and concentrated in vacuo. The residue was purified by silica-gel column chromatography (EtOAc/hexane) to afford **16** (278 mg, 756 μmol, 72%) as an amorphous yellow solid.

^{1}H NMR (400 MHz, $CDCl_3$) δ 11.73 (1H, s), 10.45 (1H, s), 7.52 (2H, d, *J* = 6.9 Hz), 7.47–7.33 (4H, m), 7.29–7.19 (4H, m), 7.13 (1H, d, *J* = 7.8 Hz), 6.97 (1H, d, *J* = 8.7 Hz), 4.93 (1H, s), 3.99 (2H, dd, *J* = 16.5, 13.3 Hz), 3.15–2.84 (4H, m); ^{13}C NMR (100 MHz, $CDCl_3$) δ 196.7, 162.4, 138.0, 136.6, 134.7, 134.1, 129.3, 129.1, 128.5, 127.6, 127.5, 127.4, 127.3, 126.1, 124.8, 119.7, 118.1, 95.4, 82.3, 59.8, 54.5, 46.0, 29.0; HRMS (ESI, *m*/*z*): $[M+H]^+$ calcd. for $C_{25}H_{22}NO_2$, 368.1646; found 368.1640.

Compound **11**

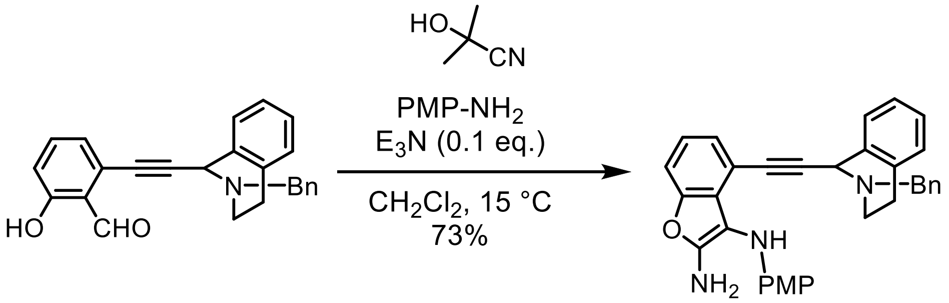

16 **11**

CAUTION: Cyanide hazard. Perform all the procedure including reaction, concentration, and purification in a well-ventilated fume hood. All waste liquids must be treated and discharged in accordance with the regulations of the organization.

To a stirred solution of **16** (63.8 mg, 174 μmol, 1.0 equiv) in CH_2Cl_2 1.7 mL (0.1 M) was sequentially added *p*-anisidine (22.8 mg, 185 μmol, 1.07 equiv), acetone cyanohydrin (16.6 μL, 182 μmol, 1.05 equiv), and triethylamine (4.84 μL, 34.7 μmol, 0.2 equiv) at 15 °C. The reaction mixture was stirred at 15 °C for 7 h, and the resulting mixture was directly concentrated in vacuo. The residue was purified by silica-gel column chromatography (EtOAc/hexane) to afford **11** as an amorphous brown solid (63.5 mg, 127 μmol, 73%).

^{1}H NMR (400 MHz, $CDCl_3$) δ 7.42–7.29 (5H, m), 7.22–7.06 (5H, m), 6.98–6.94 (1H, m), 6.88 (1H, d, J = 7.3 Hz), 6.69–6.62 (2H, m), 6.27–6.16 (2H, m), 4.68–4.67 (2H, m), 4.13 (2H, s), 3.85–3.71 (2H, m), 3.68 (3H, s), 3.05–2.91 (1H, m), 2.89–2.78 (1H, m), 2.75–2.66 (2H, m); ^{13}C NMR (100 MHz, $CDCl_3$) δ 154.0, 152.8, 148.9, 141.4, 138.8, 135.7, 134.4, 129.9, 129.3, 129.1, 128.5, 128.0, 127.9, 127.3, 127.0, 125.9, 120.4, 114.9, 114.7, 111.5, 110.3, 97.9, 90.0, 84.3, 59.6, 55.9, 54.5, 45.8, 29.3; HRMS (ESI, *m*/*z*): $[M+H]^+$ calcd. for $C_{33}H_{30}N_3O_2$, 500.2333; found 500.2338.

Compound **18**

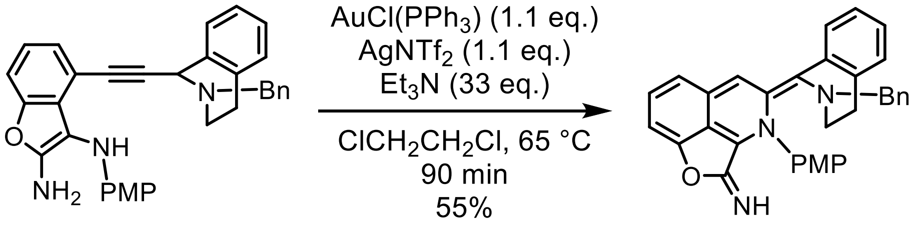

11 **18**

To a solution of $AuCl(PPh_3)$ (16.7 mg, 33.8 μmol, 1.1 equiv) in $ClCH_2CH_2Cl$ (2.5 mL, 0.01 M) was added $AgNTf_2$ (12.7 mg, 32.7 μmol, 1.1 equiv), resulting in the rapid formation of silver chloride as a white precipitation. To the resulting mixture, a solution of **11** (15.6 mg, 31.2 μmol, 1.0 equiv) and Et_3N (144 μL, 1.03 mmol, 33 equiv) in $ClCH_2CH_2Cl$ (624 μL) was added at which point the reaction mixture turned green. After stirring at 65 °C for 90 min, the resulting mixture was concentrated in vacuo. The crude residue was roughly purified by silica-gel column chromatography ($MeCN/CH_2Cl_2$) and further purified by HPLC using InertSustain C18 (GL Sciences, φ10 × 250 mm, 5 μm) under the following conditions: 0–15 min, linear gradient 65–80% MeCN in water at a flow rate of 5.0 mL/min at room temperature, UV detector λ = 254 nm. The fraction containing the desired product was concentrated *in vacuo* to afford **18** (8.59 mg, 17.3 μmol, 55%).

^{1}H NMR (400 MHz, $CDCl_3$) δ 7.30–7.28 (3H, m), 7.23 (2H, d, J = 8.7 Hz), 7.05 (1H, d, J = 8.7 Hz), 7.02–7.00 (2H, m), 6.94–6.82 (6H, m), 6.60 (1H, d, J = 6.9 Hz), 6.55 (1H, d, J = 7.3 Hz), 6.45 (1H, s), 5.42 (1H, br), 4.19 (2H, s), 3.78 (3H, s), 3.40 (2H, d, J = 4.6 Hz), 2.94 (1H, br), 2.81 (1H, br) HRMS (ESI, *m*/*z*): $[M+Na]^+$ calcd. for $C_{33}H_{27}N_3O_2Na$, 520.1996; found 520.2006.

Due to the instability of the transient intermediate **18**, a reliable ^{13}C NMR spectrum could not be obtained.

Compound **14**

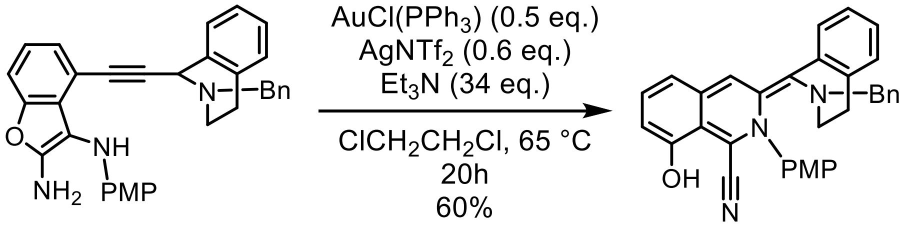

To a solution of AuCl(PPh$_3$) (7.13 mg, 14.1 µmol, 0.50 equiv) in ClCH$_2$CH$_2$Cl (31 mL, 0.001 M) was added AgNTf$_2$ (6.93 mg, 17.9 µmol, 0.60 equiv), resulting in the rapid formation of the silver chloride as a white precipitation. To the mixture, a solution of Et$_3$N (150 µL, 1.08 mmol, 34 equiv) and **11** (15.9 mg, 31.8 µmol, 1.0 equiv) in ClCH$_2$CH$_2$Cl (953 µL) was added at which point the reaction mixture turned green. After stirring at 65 °C for 20 h, the resulting reaction mixture was filtered. The filtrate was concentrated in vacuo and the crude residue was purified by PTLC (CH$_2$Cl$_2$) to afford product **14** (9.53 mg, 19.2 µmol, 60%) as a green oil.

^{1}H NMR (400 MHz, CDCl$_3$) δ 12.26 (1H, s), 8.08 (2H, d, J = 9.8 Hz), 7.43 (1H, dd, J = 8.2, 7.6 Hz), 7.36 (2H, d, J = 7.3 Hz), 7.32–7.29 (2H, m), 7.24–7.21 (1H, m), 7.20–7.16 (2H, m), 7.14 (1H, d, J = 7.3 Hz), 7.02–6.99 (2H, m), 6.94–6.91 (3H, m), 6.82 (1H, d, J = 7.9 Hz), 3.91–3.86 (1H, m), 3.84 (3H, s), 3.49–3.43 (2H, m), 3.20–3.14 (1H, m), 3.05–3.01 (1H, m), 2.87–2.83 (1H, m); ^{13}C NMR (100 MHz, CDCl$_3$) δ 171.7, 159.7, 158.9, 154.8, 140.8, 140.1, 140.0, 139.3, 136.2, 134.4, 132.9, 131.3, 131.2, 129.8, 128.4, 128.2, 127.7, 127.6, 127.0, 126.3, 125.9, 117.5, 115.3, 114.0, 110.2, 55.6, 53.9, 45.1, 30.1; HRMS (ESI, m/z): $[M+H]^+$ calcd. for $C_{33}H_{28}N_3O_2$, 498.2177; found 498.2164.

^{1}H, ^{13}C NMR spectra

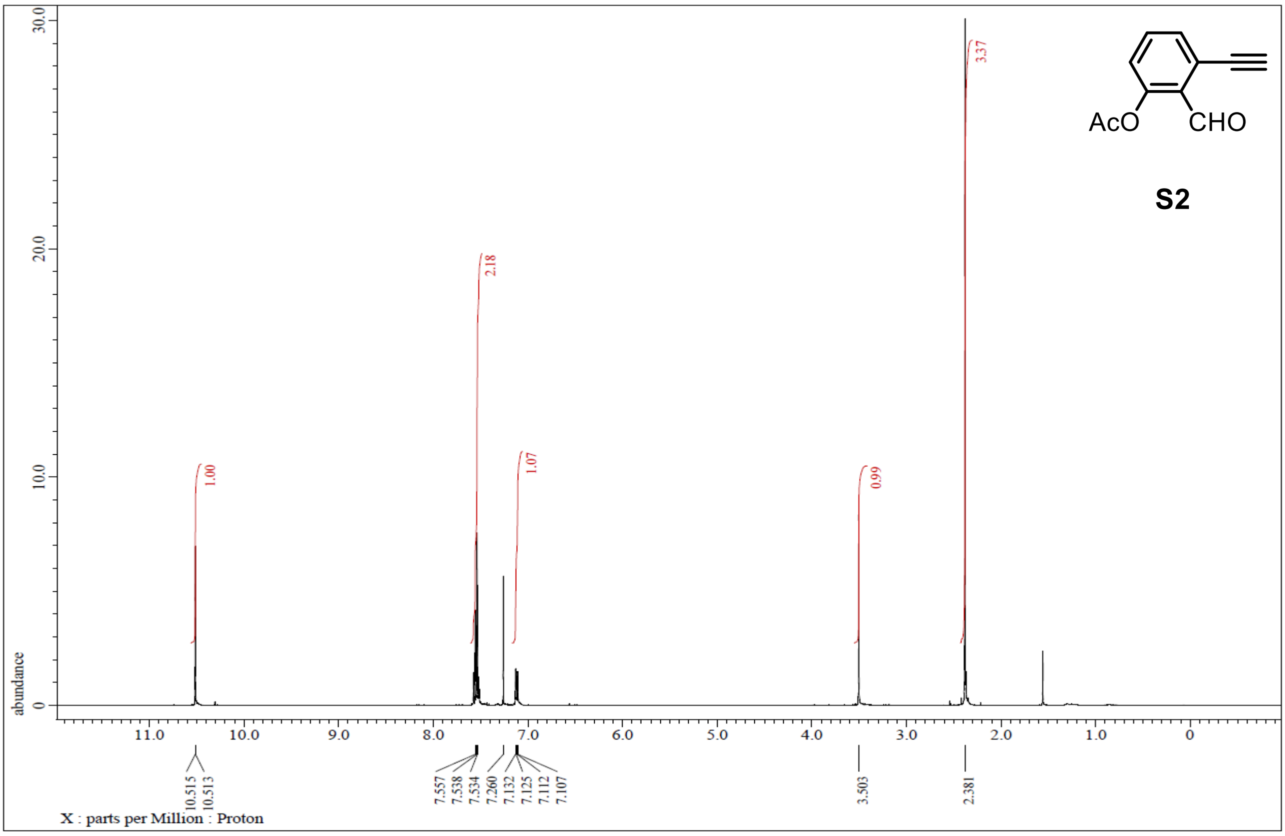

Figure S4. ^{1}H NMR spectrum (400 MHz, $CDCl_3$) of **S2**.

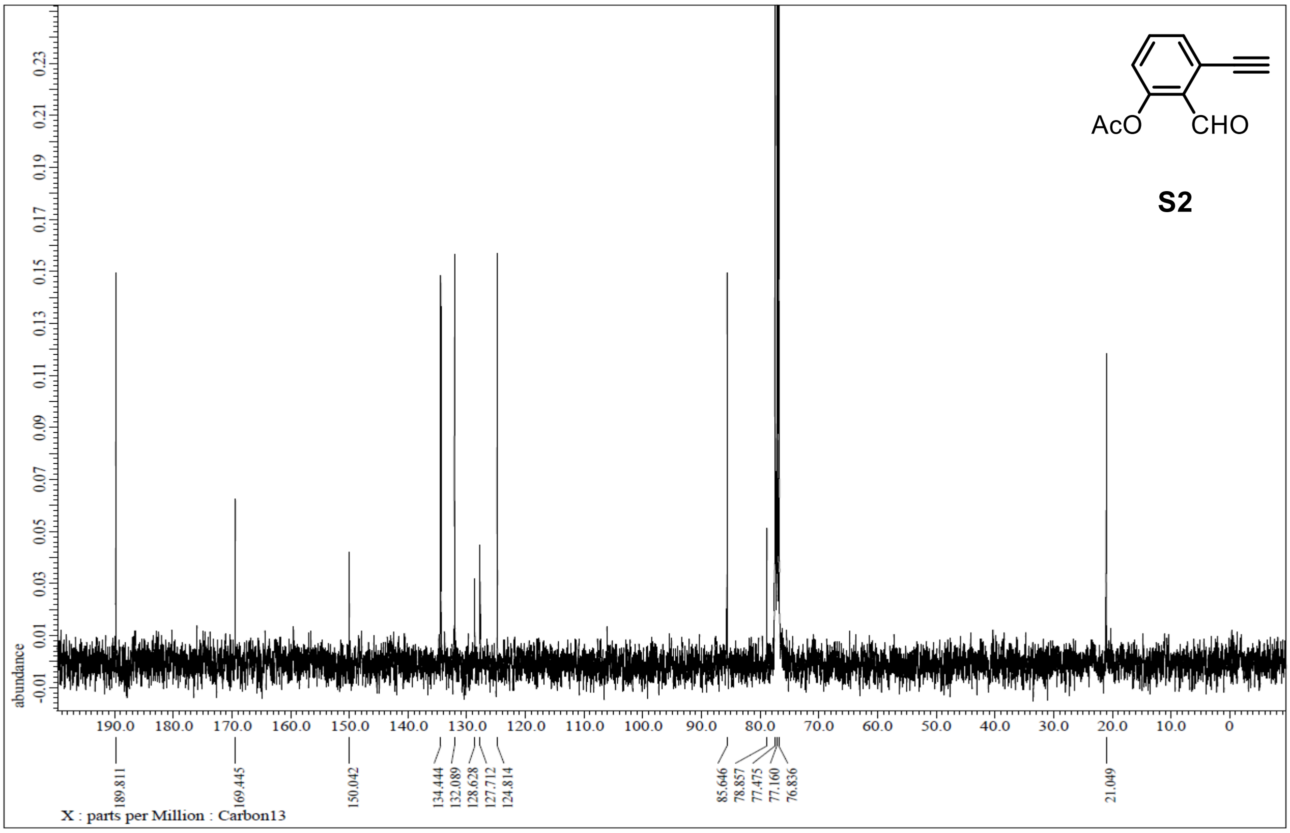

Figure S5. ^{13}C NMR spectrum (400 MHz, $CDCl_3$) of **S2**.

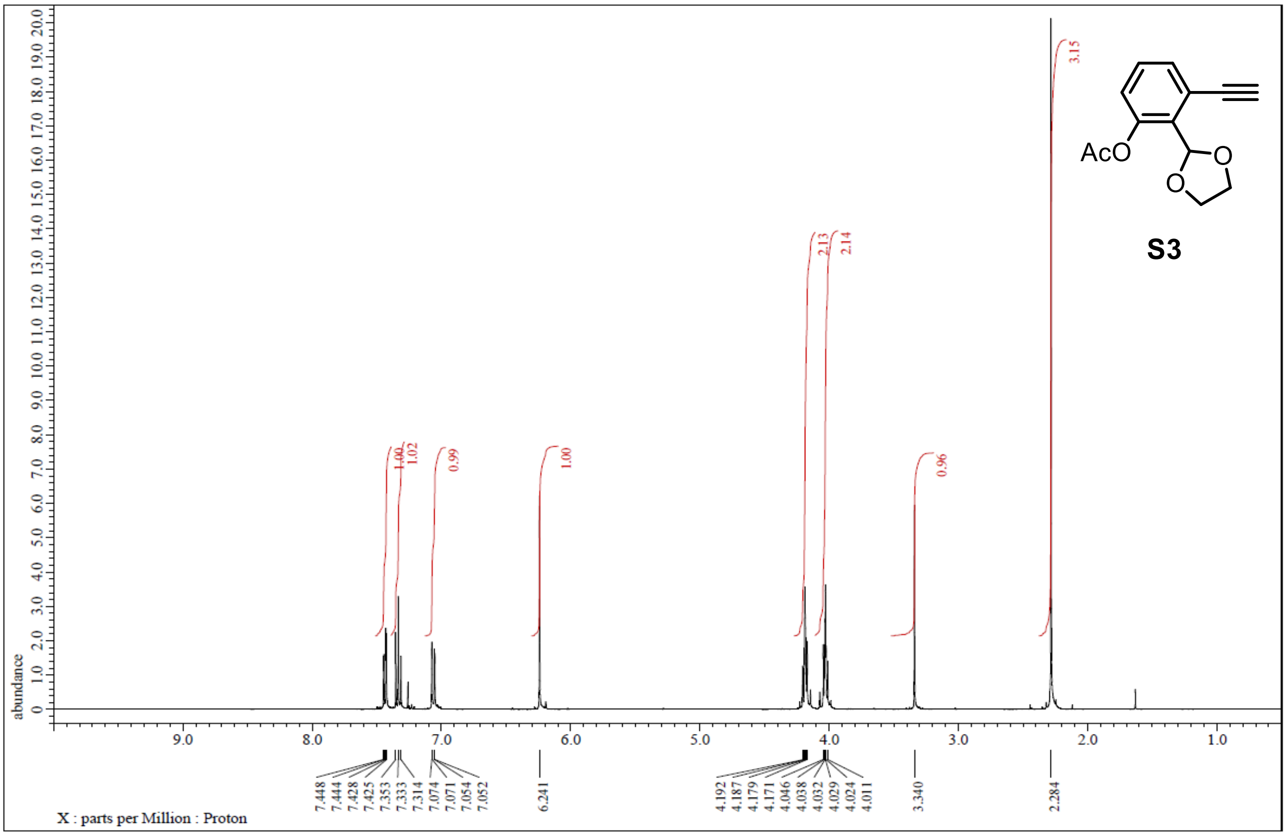

Figure S6. ^{1}H NMR spectrum (400 MHz, $CDCl_3$) of compound **S3**.

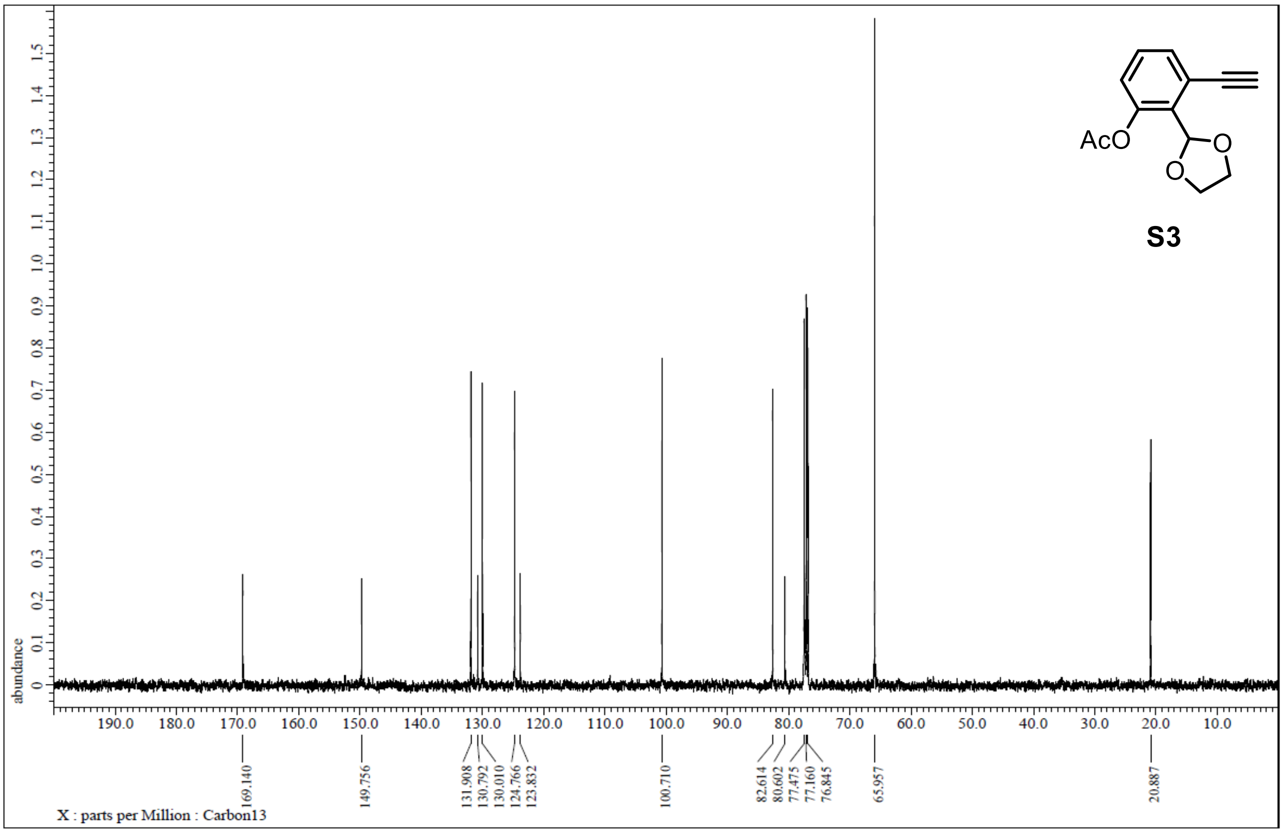

Figure S7. ^{13}C NMR spectrum (400 MHz, $CDCl_3$) of **S3**.

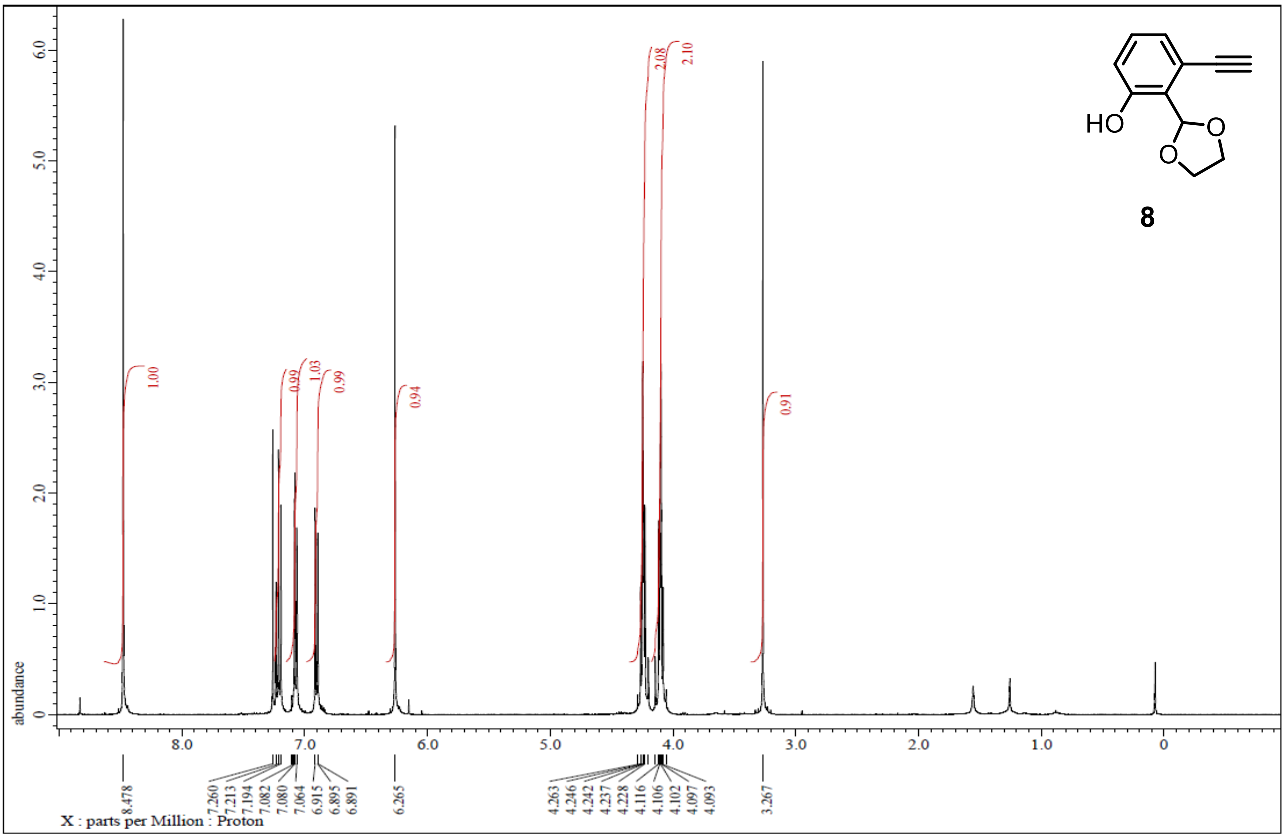

Figure S8. ^{1}H NMR spectrum (400 MHz, $CDCl_3$) of **8**.

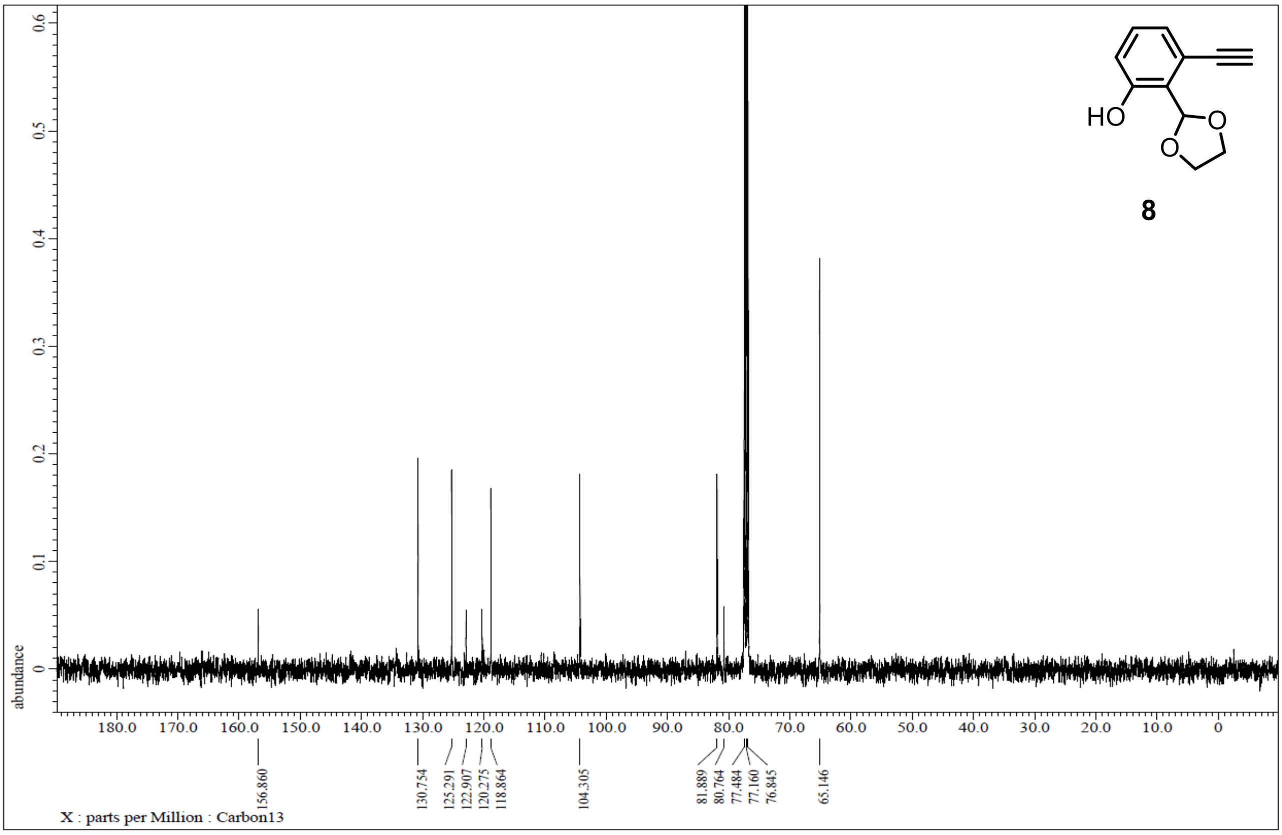

Figure S9. ^{13}C NMR spectrum (400 MHz, $CDCl_3$) of **8**.

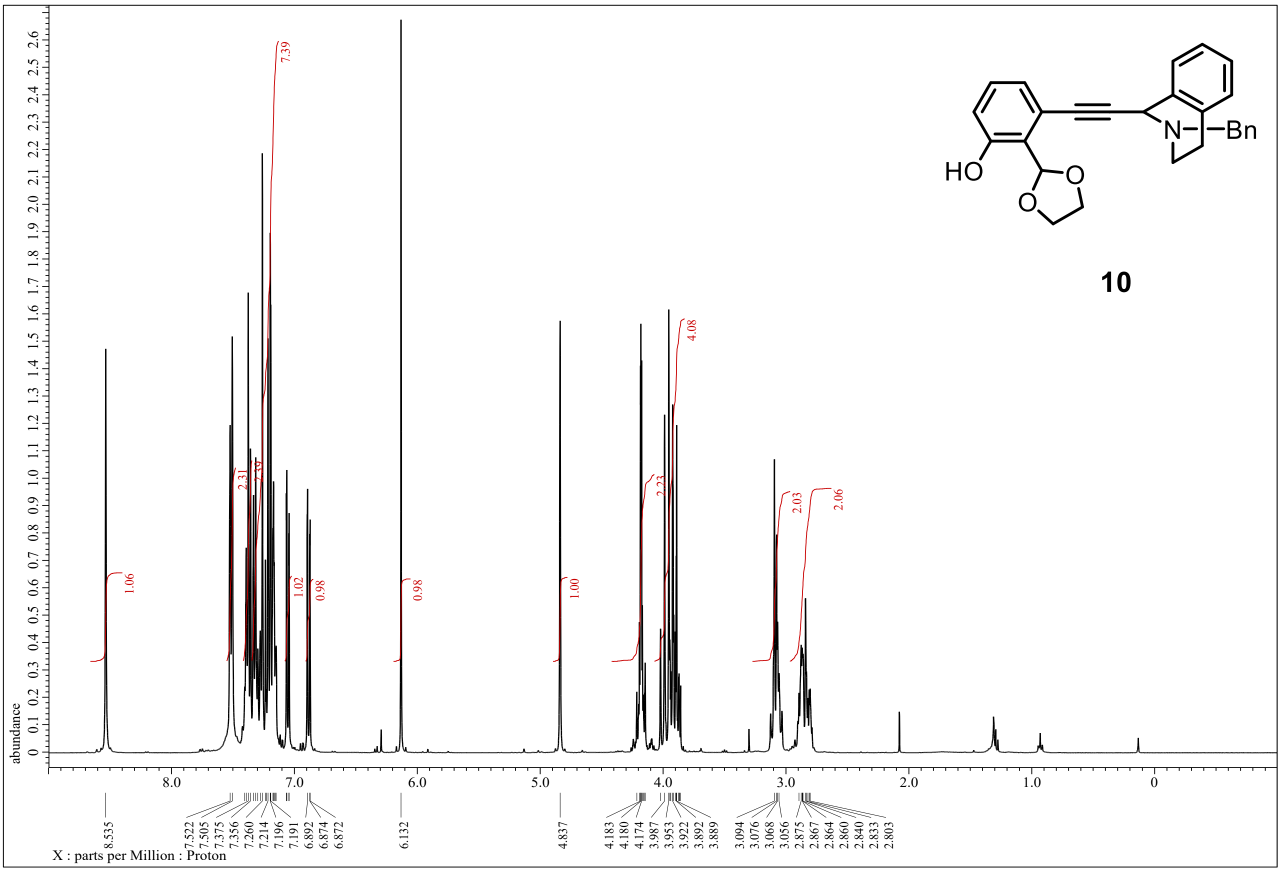

Figure S10. ^{1}H NMR spectrum (400 MHz, $CDCl_3$) of **10**.

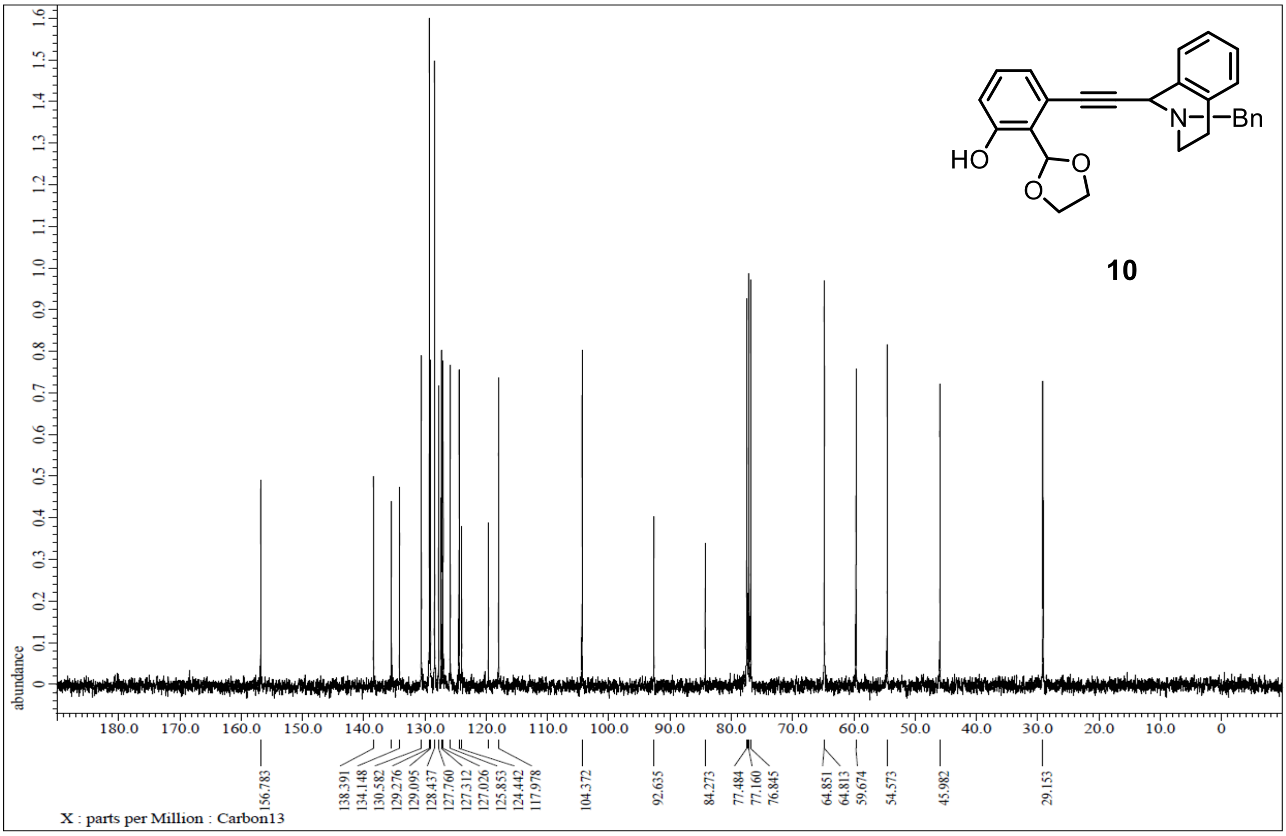

Figure S11. ^{13}C NMR spectrum (400 MHz, $CDCl_3$) of **10**.

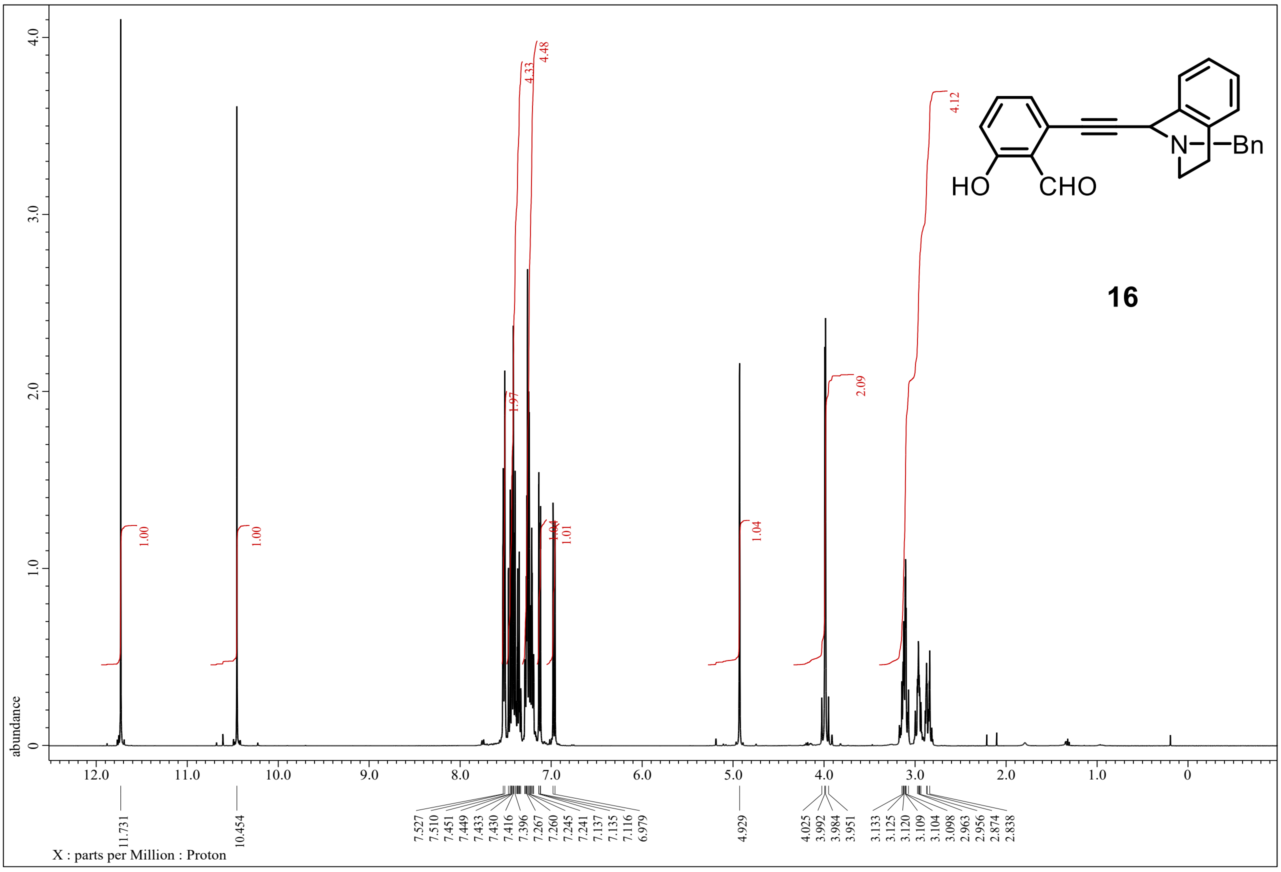

Figure S12. ^{1}H NMR spectrum (400 MHz, $CDCl_3$) of **16**.

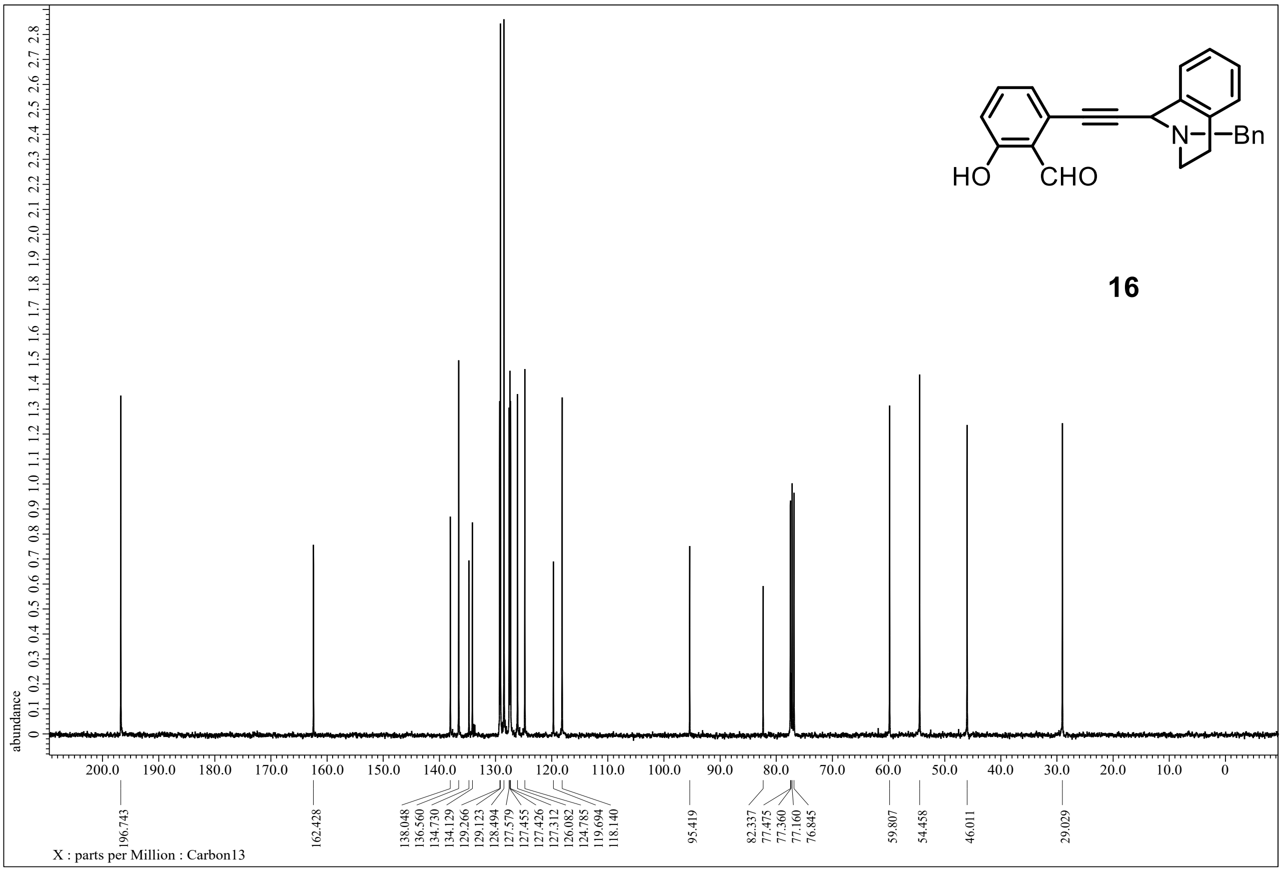

Figure S13. ^{13}C NMR spectrum (400 MHz, $CDCl_3$) of **16**.

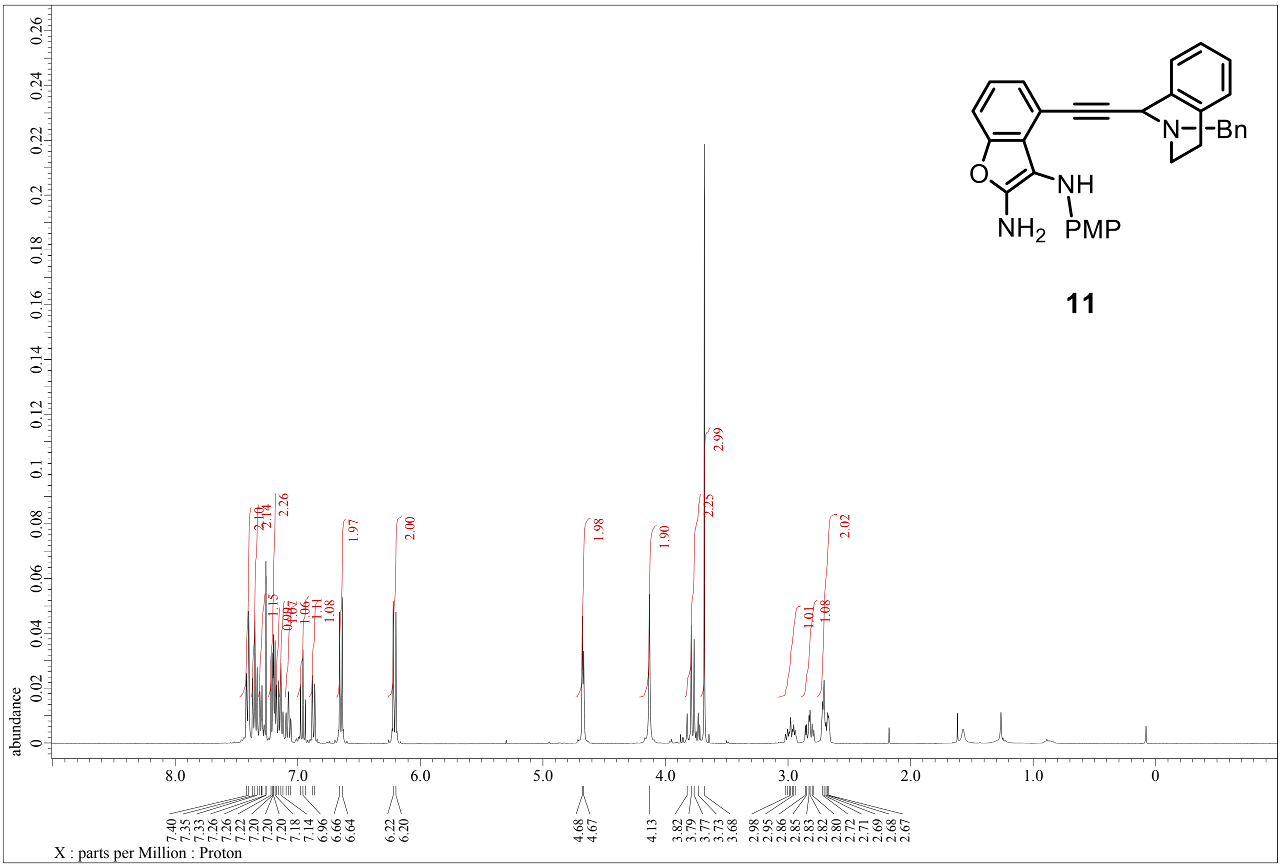

Figure S14. ^{1}H NMR spectrum (400 MHz, $CDCl_3$) of **11**.

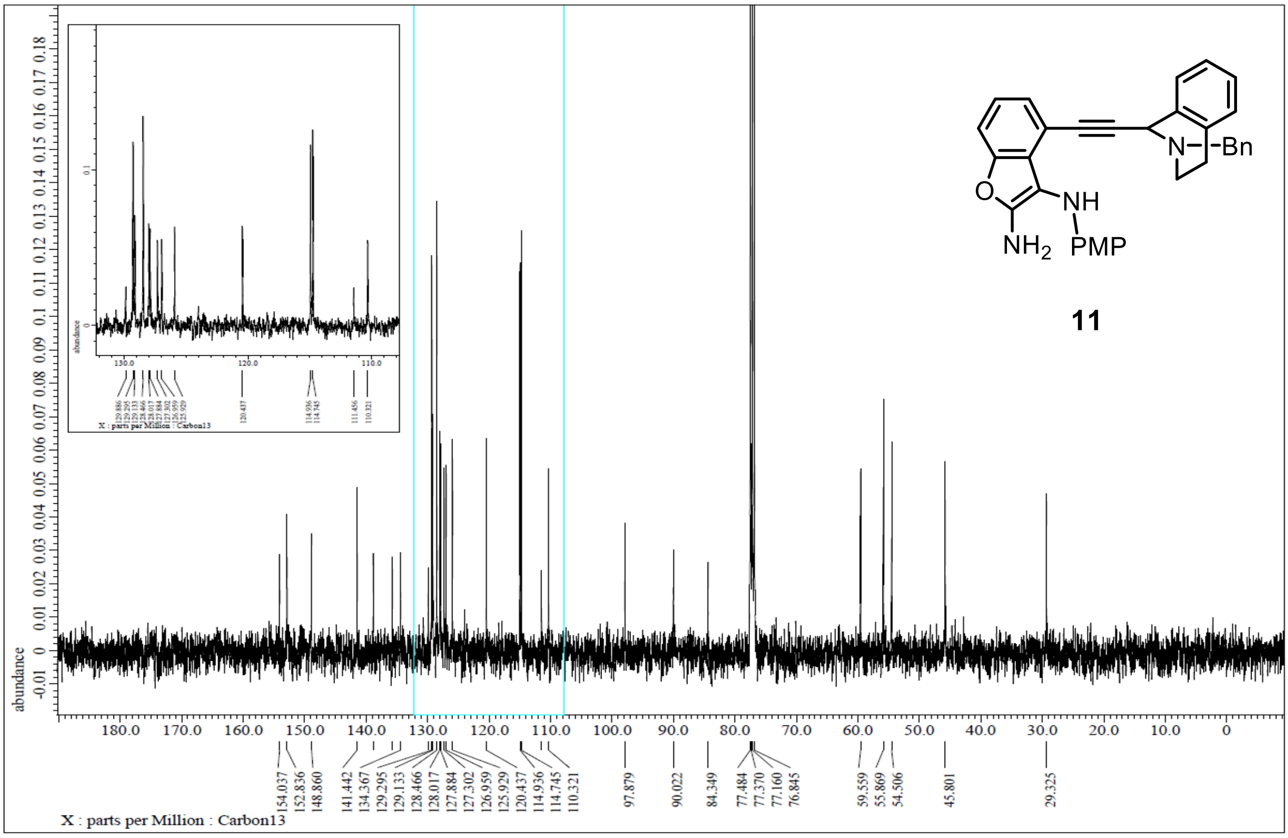

Figure S15. ^{13}C NMR spectrum (400 MHz, $CDCl_3$) of **11**.

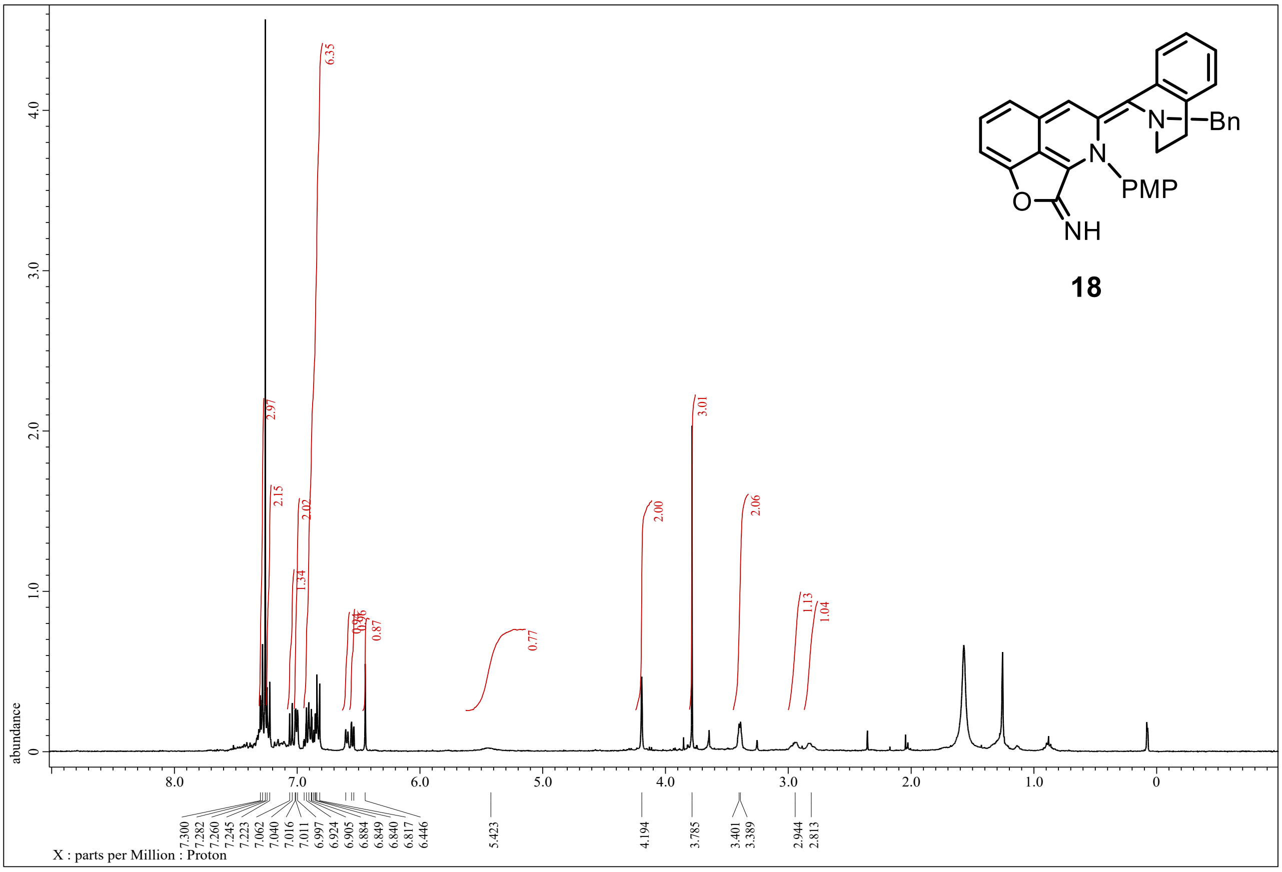

Figure S16. ^{1}H NMR spectrum (400 MHz, $CDCl_3$) of **18**.

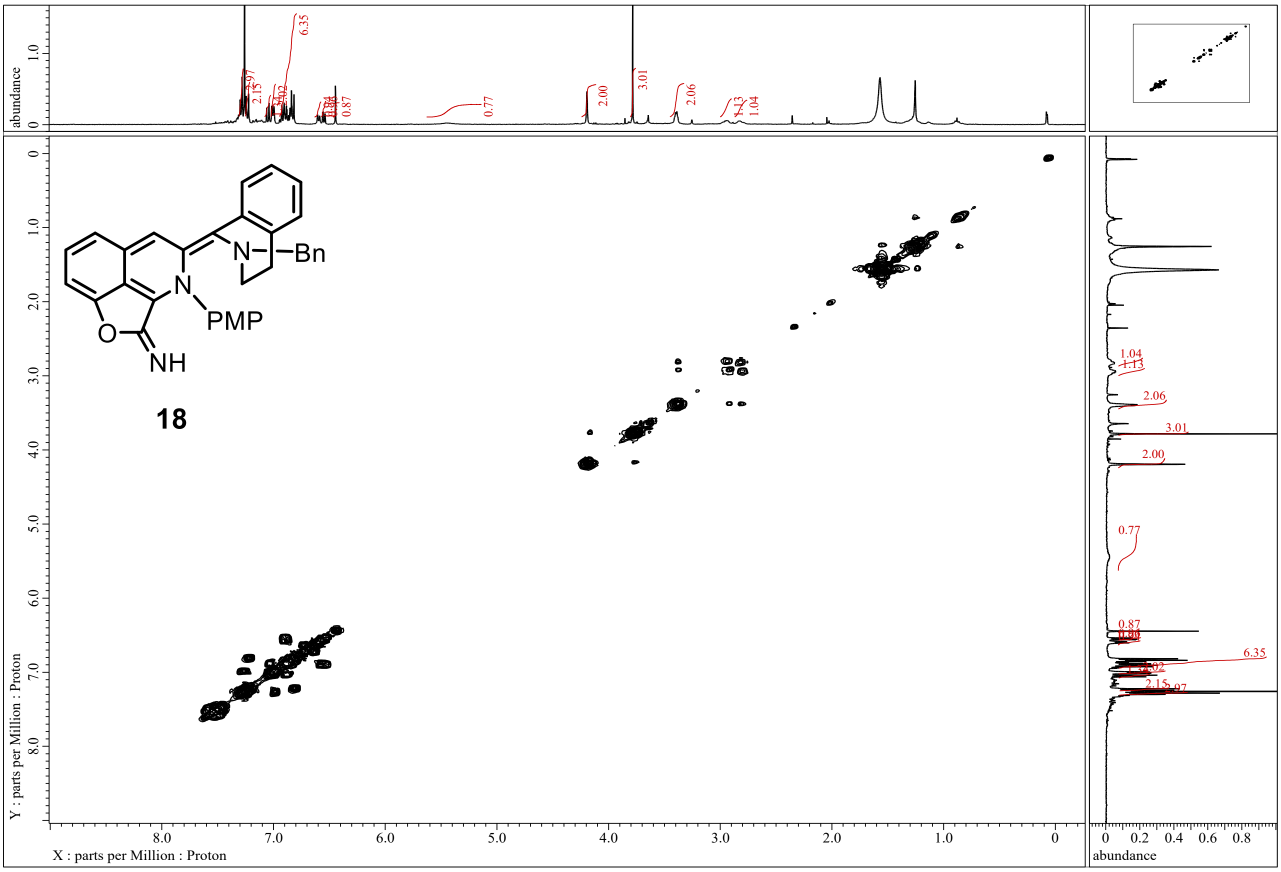

Figure S17. ^{1}H–^{1}H COSY NMR spectrum (400 MHz, $CDCl_3$) of **18**.

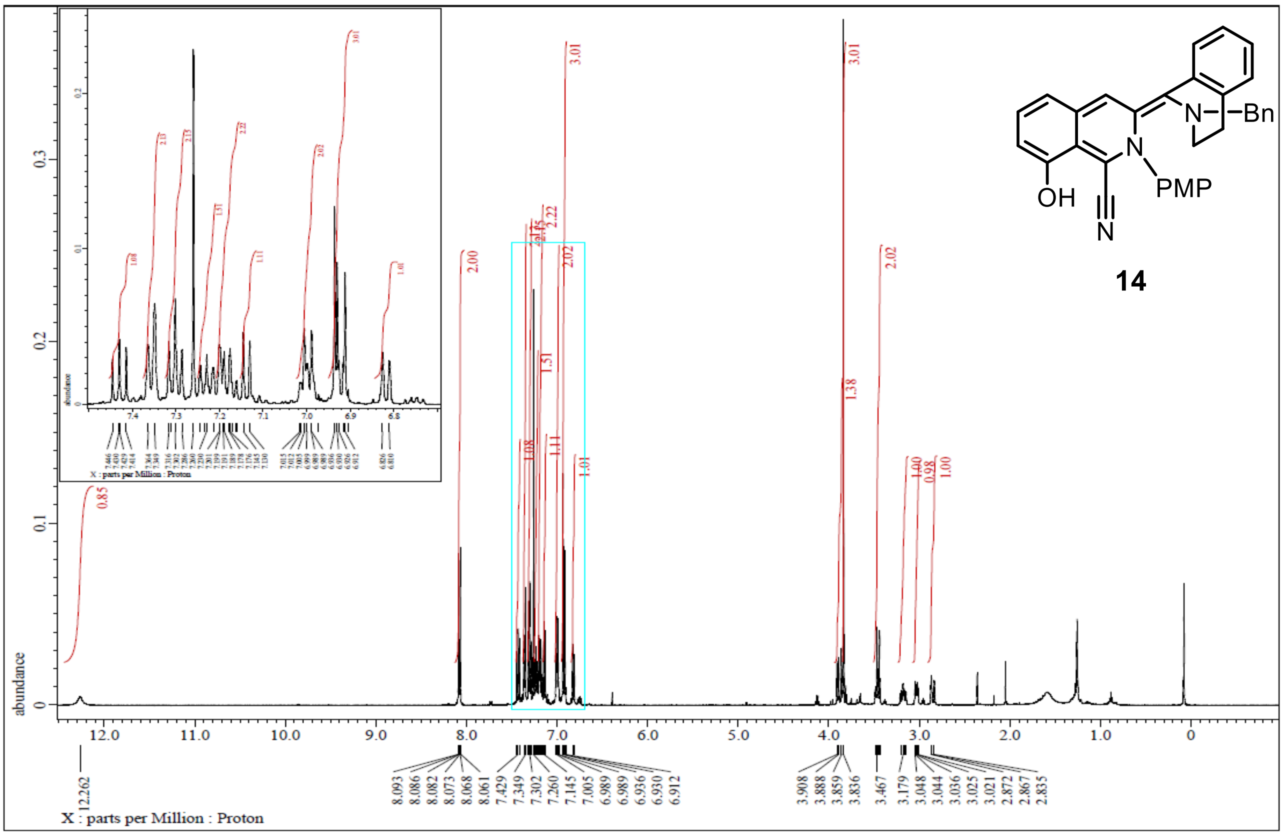

Figure S18. ^{1}H NMR spectrum (400 MHz, $CDCl_3$) of **14**.

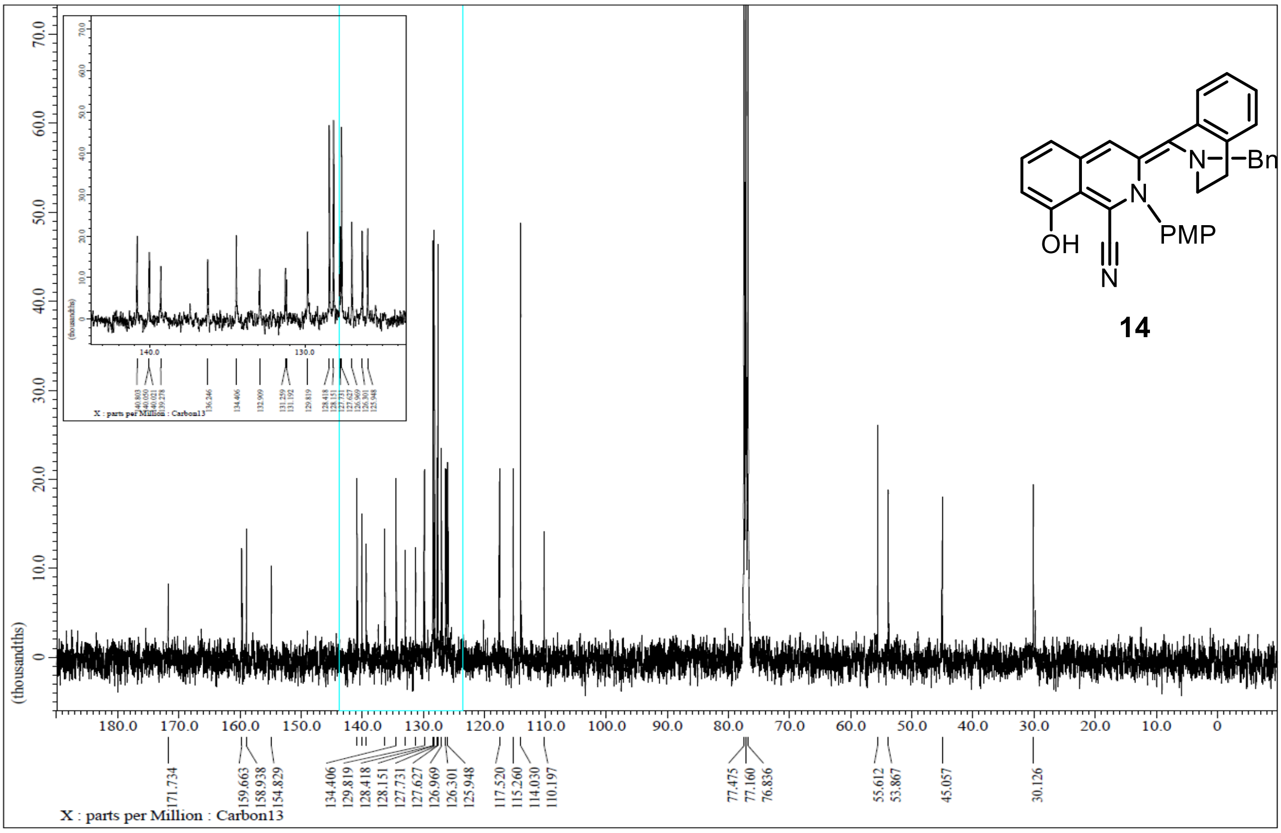

Figure S19. ^{13}C NMR spectrum (400 MHz, $CDCl_3$) of **14.**

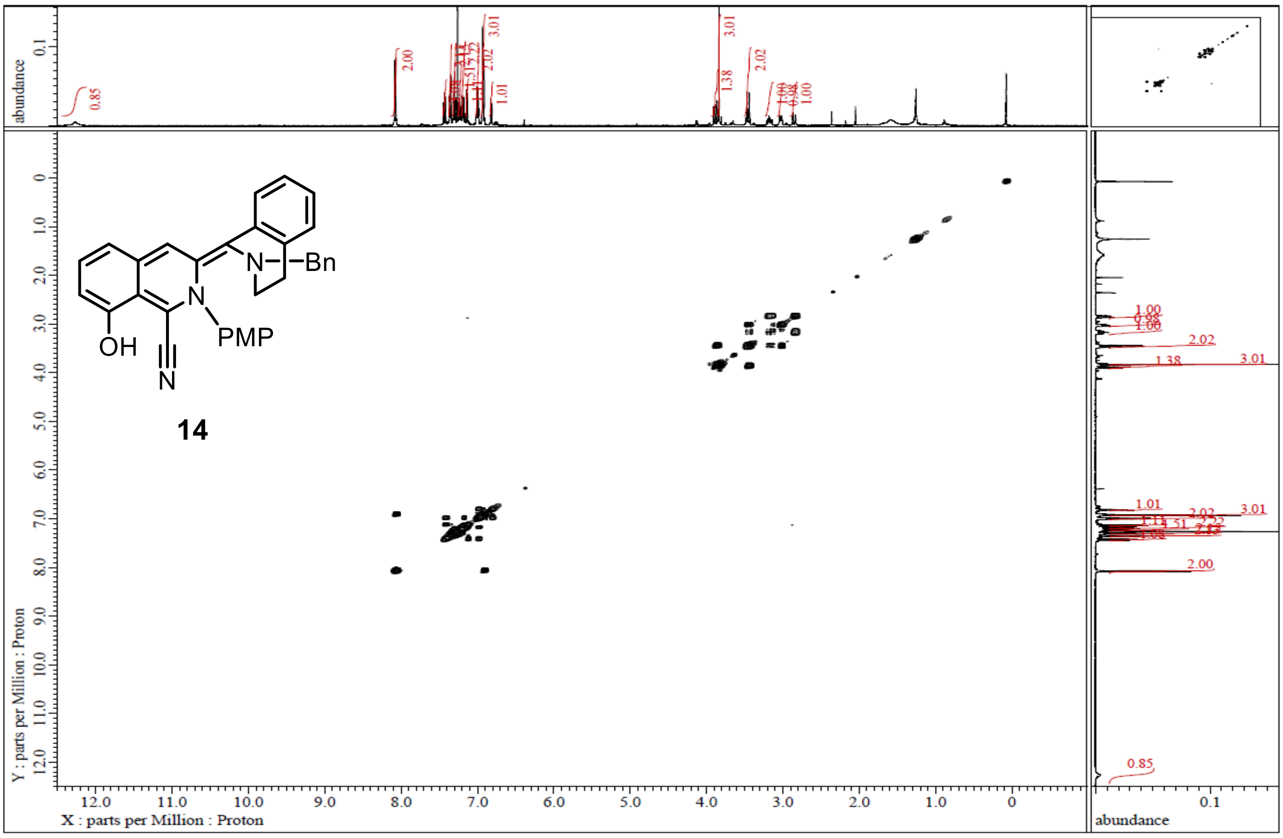

Figure S20. ^{1}H–^{1}H COSY NMR spectrum (400 MHz, $CDCl_3$) of **14**.

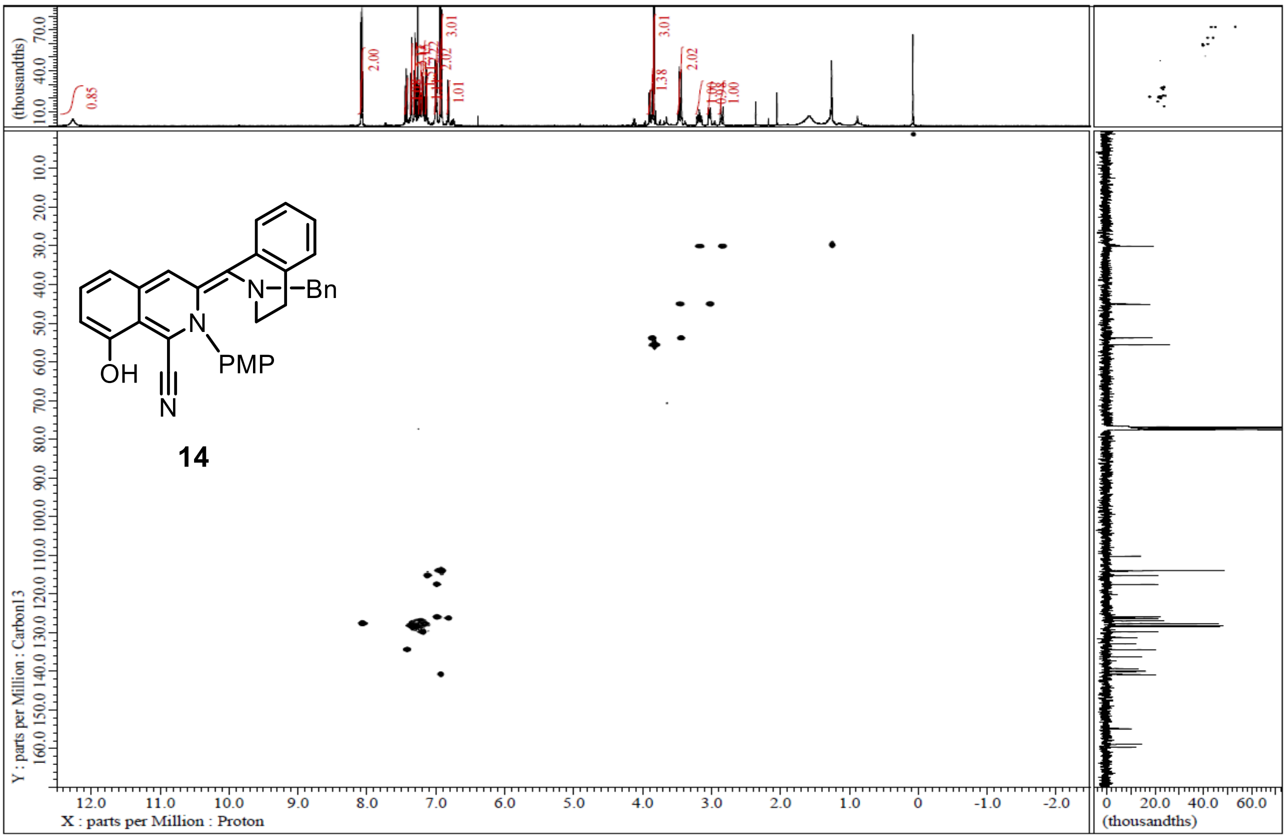

Figure S21. ^{1}H–^{13}C HSQC NMR spectrum (400 MHz, $CDCl_3$) of **14**.

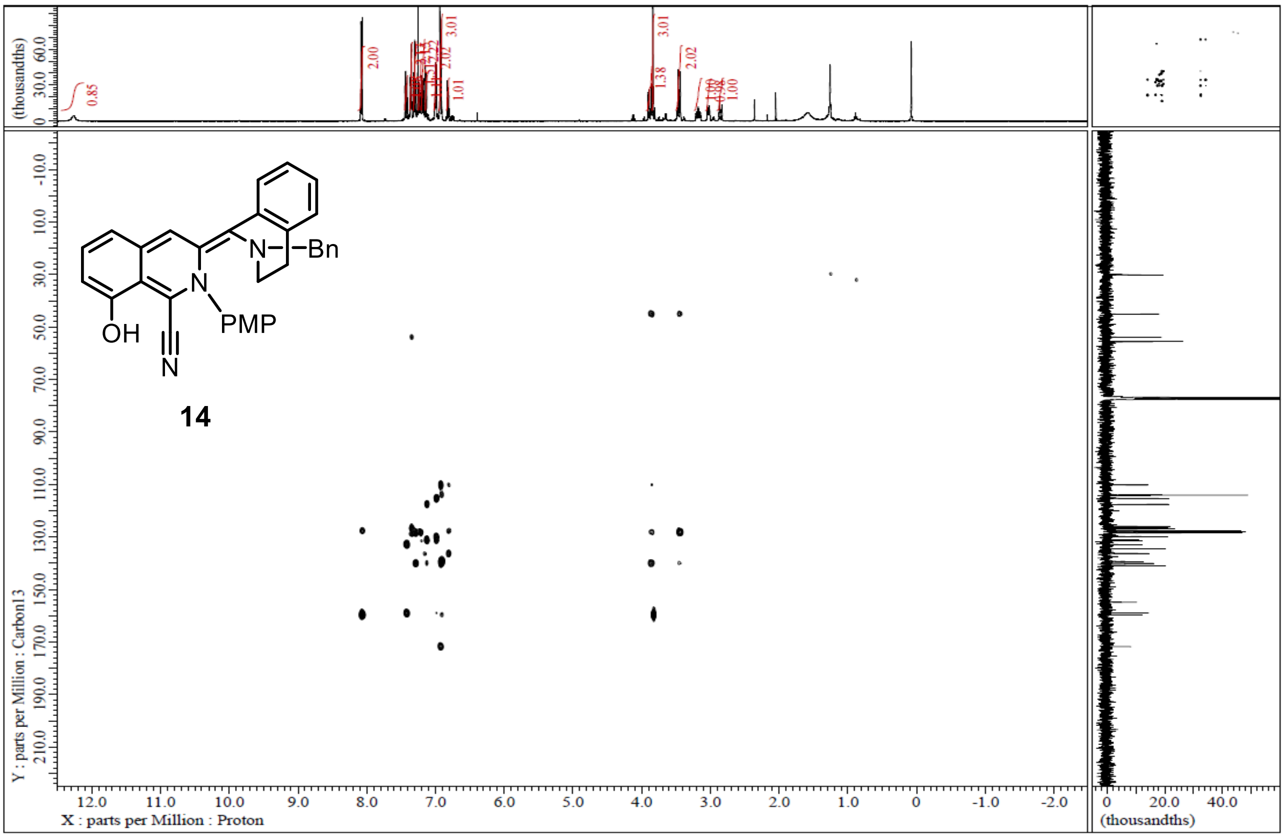

Figure S22. ^{1}H–^{13}C HMBC NMR spectrum (400 MHz, $CDCl_3$) of **14**.

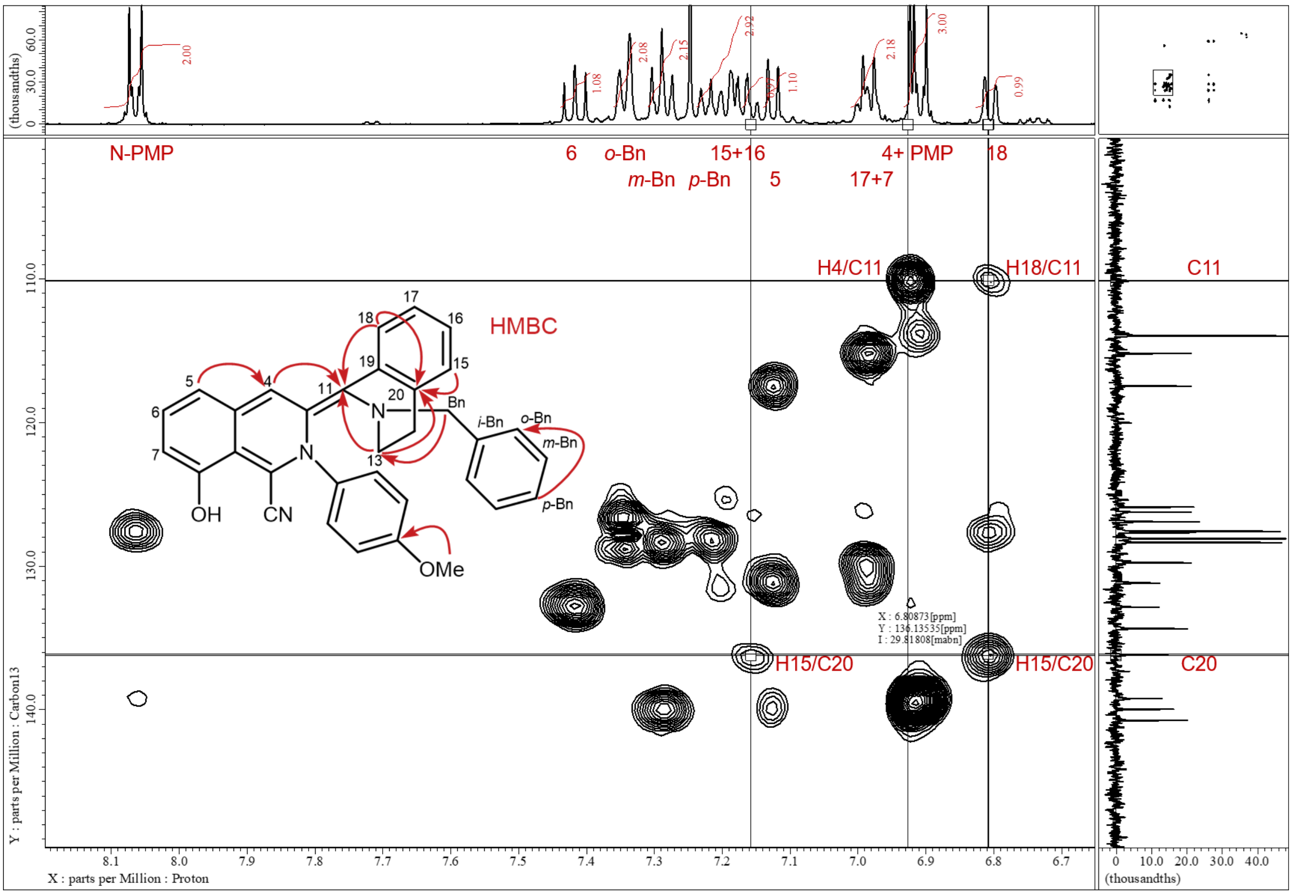

Figure S23. A closer look of ^{1}H–^{13}C HMBC NMR spectrum (400 MHz, $CDCl_3$) of **14**.

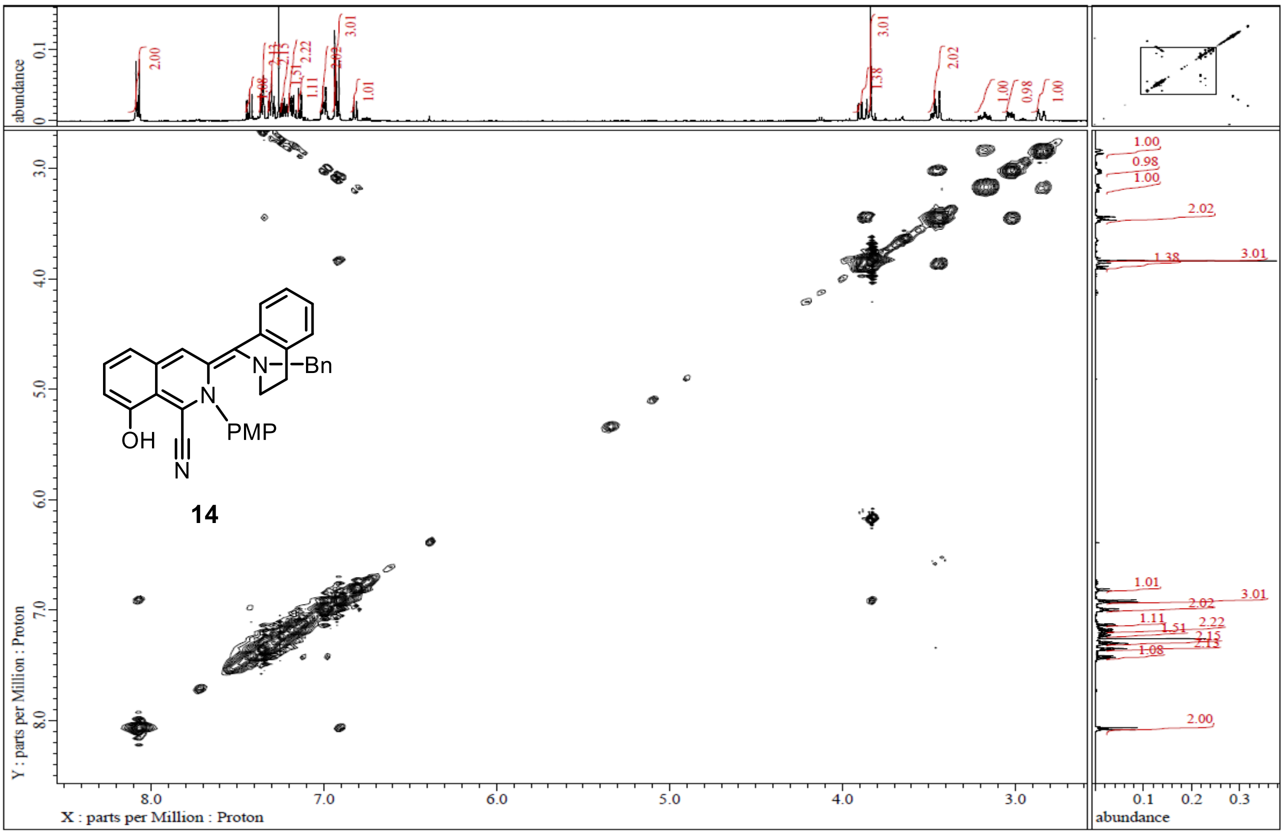

Figure S24. ^{1}H–^{1}H NOESY NMR spectrum (400 MHz, $CDCl_3$) of **14**.

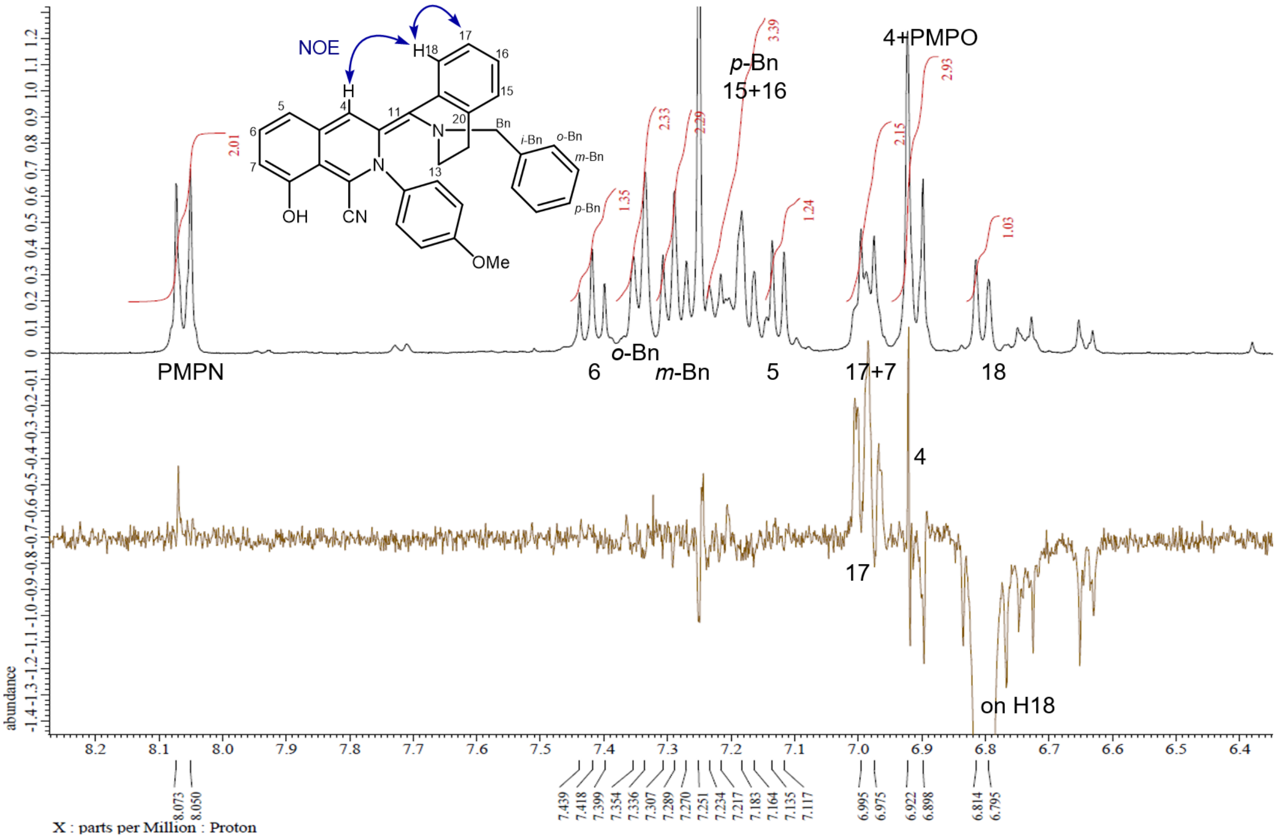

Figure S25. Difference NOE spectrum (400 MHz, $CDCl_3$) of **14**. Irradiation on 6.805 ppm (H18).

X-ray crystallographic analysis

Crystallographic data of compound 16.

All structures were refined. Crystals of compound **16** were mixed with Paraber (Hampton Research) oil and spread them onto 4 × 4 mm^2 polyimide flat-faced plate. The samples were pasted onto the poly ether ether ketone film fixed on the sample holder.

The sample holder was mounted in a specialized sample-pin holder equipped on the stage of beamline 2 at experimental hutch 3 of SACLA XFEL facility. The photon energy of the XFEL and the beam size at the sample plane were adjusted to 15.0 keV (i.e. λ = 0.827 Å) and ≈ 1 μm, respectively. The duration and repetition rate of the pulses were 7 fs and 30 Hz, respectively. The pulse energy was ≈ 140 μJ or ≈ 200 μJ per pulse. The sample plate was scanned in 2D direction over the entire plate as the XFEL beam was exposed every 10 μm horizontally and 15 μm vertically. Diffraction images were recorded by a MX300-HS charge-coupled device detector (Rayonix) fixed 50 mm downward to the sample holder. All data acquisition was conducted at room temperature.

CCD frames showing Bragg spots were first identified using a diffraction data-processing program, DIALS[4] with the option *force_2d=True*, for *dials.find_spots*. Frames showing diffraction patterns from a single crystal were identified based on the number of diffraction spots in a frame. Frames having less than 5 diffraction spots or more than 500 diffraction spots were excluded in this step. The remaining frames were used as the hit frames and proceeded using the CrystFEL suite [5], version 0.9.1. Indexing of the hit frames was first tried with the option defined as *--mosflm-nolatt-nocell*. 38 frames out of 1638 frames (2.32%) were successfully indexed and some candidates of unit cell parameters were proposed. Indexing was repeated with an option of *--xgandalf* by specifying unit cell parameters as (a, b, c, α, β, γ) = (9.37, 13.88, 30.35, 90, 90, 90), which was the most plausible considered from the molecule size. After the optimization of the various indexing parameters, 6189 frames out of 13487 frames (45.89%) were successfully indexed and integrated under the condition defined as *--threshold=0*, *--min-pix-count=2*, *--local-bg-radius=3*, *--min-snr=4.5*, and *--int-radius=3,4,7*. Integrated intensities were merged by *process_hkl* in the CrystFEL suite. Statistics of the merged data were calculated by *compare_hkl* and *check_hkl* in the CrystFEL suite. The resolution limit was estimated 0.78 Å from these statistics. The structure was solved by a direct method with the SHELXT software program[6] and refined by full-matrix least-squares on F^2 using the SHELXL-2018/3 software program[7].

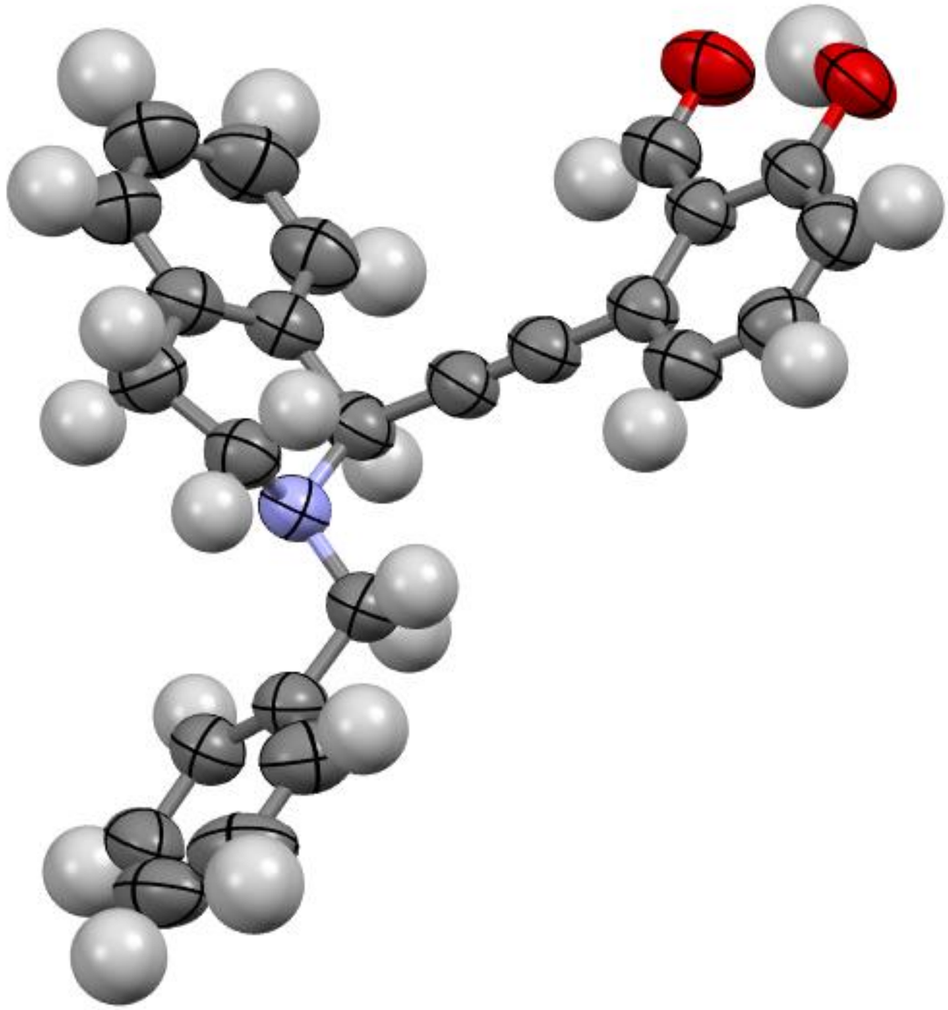

Figure S25. The scheme of **16** with 50% thermal ellipsoid probability levels. The hydrogen atoms are shown as circles for clarity.

Table S3. Crystal data and structure refinement for **16**.

Empirical formula	$C_{25}H_{21}NO_2$
Formula weight	367.43
Temperature/K	288
Wavelength/Å	0.827
Crystal system	Orthorhombic
Space group	*P*bca
a/Å	13.880
b/Å	9.370
c/Å	30.350
α/°	90
β /°	90
γ /°	90
Volume/Å^3	3947.2
Z	8
Calculated density (g·cm^{-3})	1.237
Absorption coefficient (mm^{-1})	0.110
F(000)	1552.0
Crystal size/mm	0.005 × 0.005 × 0.005
2θ range for data collection/°	2.313 to 31.996
Limiting indices	−17 ≤ h ≤ 17, −11 ≤ k ≤ 12, −38 ≤ l ≤ 38
Reflections collected	8177
Independent reflections	4346 [*R*(int) = 0.0979]
Completeness to θ_{max}/%	99.9
Data / restraints / parameters	4346/0/256
Finale *R* indices [*I*>2σ(*I*)]	R_1 = 0.2036, wR_2 = 0.3397
R indices (all data)	R_1 = 0.2123, wR_2 = 0.3807
Goodness-of-fit on F^2	1.277
Deposit number CCDC	2352718

References

1. Jiang, G.-J.; Zheng, Q.-H.; Dou, M.; Zhuo, L.-G.; Meng, W.; Yu, Z.-X. *J. Org. Chem.* **2013**, *78*, 11783–11793.
doi:10.1021/jo4018183
2. Takaba, K.; Maki-Yonekura, S.; Inoue, I.; Tono, K.; Hamaguchi, T.; Kawakami, K.; Naitow, H.; Ishikawa, T.; Yabashi, M.; Yonekura, K. *Nat. Chem.* **2023**, *15*, 491–497.
doi:10.1038/s41557-023-01162-9
3. Hsu, D.-S.; Liou, C.-Y. *Org. Biomol. Chem.* **2018**, *16*, 4990–4995.
doi:10.1039/C8OB01207E
4. Clabbers, M. T. B.; Gruene, T.; Parkhurst, J. M.; Abrahams, J. P.; Waterman, D. G. *Acta Crystallogr. D Struct. Biol.* **2018**, *74*, 506–518.
doi:10.1107/S2059798318007726
5. White, T. A.; Mariani, V.; Brehm, W.; Yefanov, O.; Barty, A.; Beyerlein, K. R.; Chervinskii, F.; Galli, L.; Gati, C.; Nakane, T.; Tolstikova, A.; Yamashita, K.; Yoon, C. H.; Diederichs, K.; Chapman, H. N. *J. Appl. Crystallogr.* **2016**, *49*, 680–689.
doi:10.1107/S1600576716004751
6. Sheldrick, G. M. *Acta Crystallogr. Sect. A Found. Crystallogr.* **2015**, *71*, 3–8.
doi:10.1107/S2053273314026370
7. Sheldrick, G. M. *Acta Crystallogr. Sect. C Struct. Chem.* **2015**, *71*, 3–8.
doi:10.1107/S2053229614024218
